# Antigen‐Targeting Inserted Nanomicelles Guide Pre‐Existing Immunity to Kill Head and Neck Cancer

**DOI:** 10.1002/advs.202410629

**Published:** 2025-03-17

**Authors:** Lizhuo Zhang, Qingqing Feng, Chuanming Zheng, Yuanqiang Li, Xinyang Ge, Tiefeng Jin, Gaofeng Hu, Zhuo Tan, Jiafeng Wang, Jiajie Xu, Liehao Jiang, Dan Wang, Zhangguo Ying, Xiao Zhao, Keman Cheng, Qinglin Li, Minghua Ge

**Affiliations:** ^1^ Otolaryngology & Head and Neck Center Cancer Center Department of Head and Neck Surgery Zhejiang Provincial People's Hospital (Affiliated People's Hospital) Hangzhou Medical College Hangzhou 310014 China; ^2^ Zhejiang Provincial Clinical Research Center for Head & Neck Cancer Hangzhou 310014 China; ^3^ Zhejiang Key Laboratory of Precision Medicine Research on Head & Neck Cancer Hangzhou 310014 China; ^4^ Zhejiang Provincial Key Laboratory of Pancreatic Disease Hangzhou 310006 China; ^5^ Zhejiang Cancer Hospital Hangzhou 310005 China; ^6^ Chinese Academy of Medical Sciences & Peking Union Medical College Beijing 100193 China; ^7^ Cancer Centre Faculty of Health Sciences University of Macau Macau SAR 999078 China; ^8^ CAS Key Laboratory for Biomedical Effects of Nanomaterials and Nanosafety CAS Center for Excellence in Nanoscience National Center for Nanoscience and Technology Beijing 100190 China; ^9^ Department of Thyroid and Breast Surgery Zhejiang Provincial People's Hospital Bijie Hospital Bijie 551700 China

**Keywords:** cancer immunotherapy, head and neck cancer, nanomicelle, preS1, reprogramming of “anti‐viral immunity” into “anti‐tumor immunity”

## Abstract

A significant challenge in cancer therapy is the identification of suitable targets that are specifically and uniformly expressed across heterogeneous tumors. The efficacy of pre‐existing antiviral immunity in tumor treatment is limited by the absence of corresponding targets. This study develops a novel platform of antigen‐targeted inserted nanomicelles, preS1 (an antigen of hepatitis B virus)‐pHLIP nanomicelles, in which tumor‐targeting nanomicelles release antigens that label tumor tissue for pre‐existing immunity‐mediated lysis in situ. In animal models of head and neck cancers, including head and neck squamous cell carcinoma and anaplastic thyroid cancer, preS1‐pHLIP nanomicelles effectively inhibited tumor growth, recurrence, and metastasis in animals pre‐immunized with preS1. This therapeutic effect is associated with an increase in the proportion of preS1‐specific B cells and activated tumor‐specific T cells within the tumor microenvironment. Overall, this work has engineered a nanomicelle that can disguise tumor cells as viruses and achieve tumor killing through the pre‐existing antiviral immune response. This strategy presents a novel approach for treating tumors with ambiguous therapeutic target profiles.

## Graphical abstract

This study introduces a tumor‐targeted nanomicelle platform (preS1‐pHLIP) that exploits pre‐existing antiviral immunity to combat heterogeneous cancers. By delivering viral antigens to label tumors as virus‐like targets, the nanomicelles activate antiviral B and T cells, triggering in situ tumor lysis. In aggressive head and neck cancer models, this approach suppresses tumor growth, recurrence, and metastasis, offering a breakthrough for treating tumors lacking defined therapeutic targets.

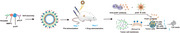

## 1 Introduction

Identifying optimal therapeutic targets remains a critical challenge in advancing cancer treatments, as tumor heterogeneity and evolutionary dynamics introduce complexities that limit the efficacy of targeted approaches. Many therapeutic targets are not uniquely expressed in tumor cells, and their expression can vary significantly across different regions and cellular subpopulations within the tumor microenvironment.^[^ ^1^ ^]^ Additionally, the continuous evolution of tumors under selective pressure from treatment often leads to alterations in target expression, impeding long‐term therapeutic success.^[^ ^2^ ^]^ For certain rare cancers and tumor subtypes, limited research on target expression further constrains the development of effective targeted therapies, posing an urgent need for innovative solutions.

Existing approaches to overcome these challenges include gene editing, small‐molecule inhibitors, and monoclonal antibody therapies aimed at modulating target expression or blocking key signaling pathways.^[^ ^3^ ^]^ While promising, these methods face notable challenges, including suboptimal delivery efficiency, potential off‐target effects, and the emergence of drug resistance.^[^ ^3d^ ^]^ Novel therapeutic strategies have started exploring the deliberate introduction of targets within the tumor environment, leveraging synthetic biology to enhance immunotherapy efficacy. For instance, using engineered bacteria capable of selectively colonizing immune‐privileged tumor sites, antigens recognizable by CAR‐T cells can be locally released, thus directing the immune response specifically to the tumor core.^[^ ^4^ ^]^ However, this approach necessitates sophisticated CAR‐T cell design to optimize both efficacy and safety, underscoring the complexity of targeted immune interventions.

An intriguing and underexplored resource in antitumor immunity lies within the body's pre‐existing antiviral immune repertoire, such as the immune memory induced by vaccinations (e.g., hepatitis B).^[^ ^5^ ^]^ This immune memory is capable of rapidly generating neutralizing antibodies upon encountering viral antigens, but its specificity to viral targets limits its utility against tumor cells.^[^ ^6^ ^]^ Transforming this antiviral immune capacity into a potent antitumor response could open novel therapeutic avenues. By devising strategies to repurpose antiviral immunity for tumor recognition, there is potential to expand the body's natural immune arsenal, potentially offering a new frontier in cancer immunotherapy.

**Figure 1:**
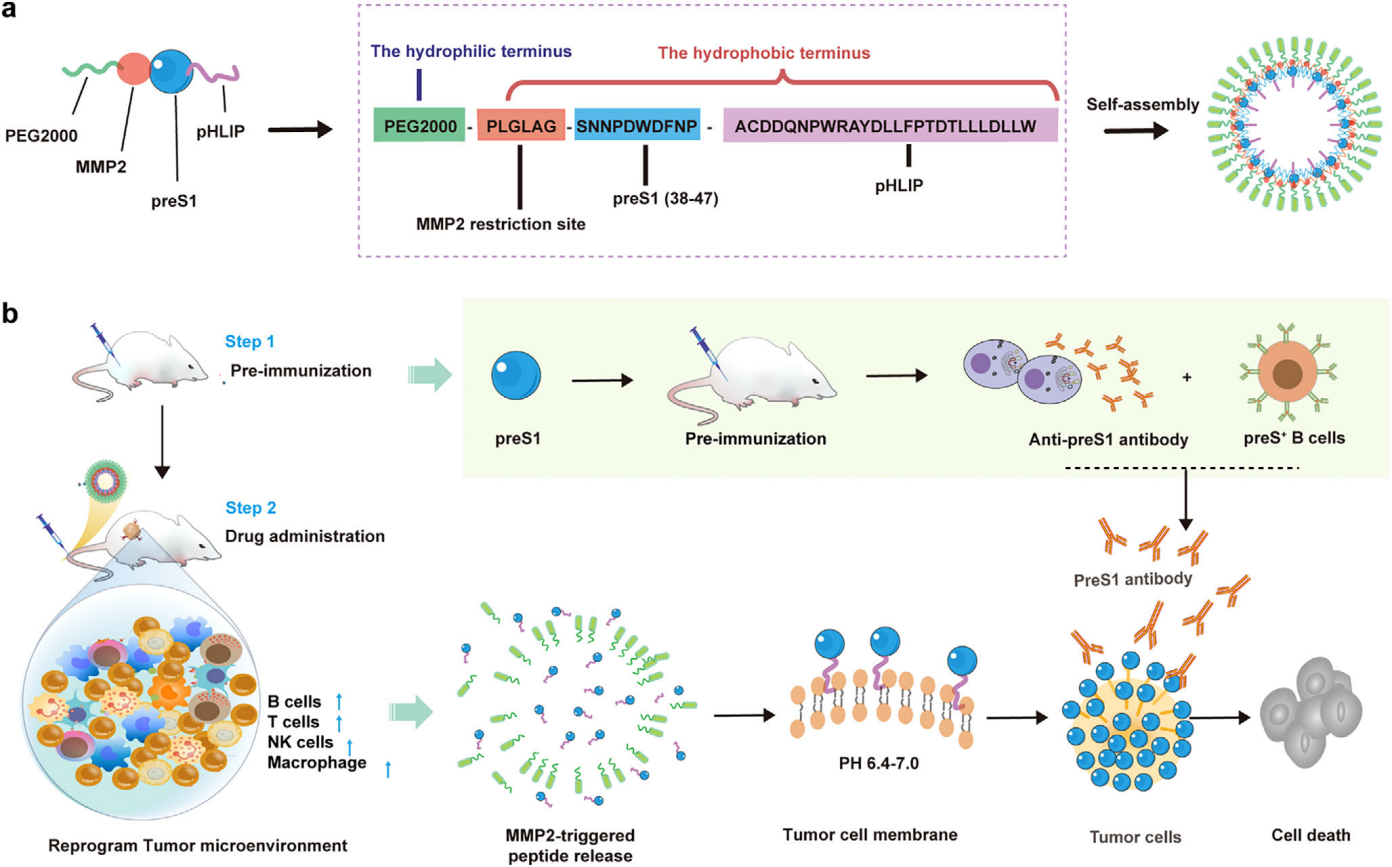
Schematic illustration of preS1‐pHLIP NMs self‐assembled polypeptide design and tumor killing strategy. a) Design of self‐assembled preS1‐pHLIP NMs. PreS1‐pHLIP NMs result from the self‐assembly of PEG2000‐PLGLAG (MMP2 restriction site)‐preS1_(38‐47)_‐pHLIP molecules, constituted of a hydrophilic PEG2000 region, and a hydrophobic region made of the fusion peptide PLGLAG (MMP2 restriction site)‐preS1_(38‐47)_‐pHLIP. b) In vivo tumor‐targeting based on preS1‐pHLIP release in the tumor microenvironment (TME) and subsequent activation of memory antiviral immunity against preS1. Upon intravenous injection of preS1‐pHLIP NMs, MMP2 activity in the tumor microenvironment releases preS1‐PHLIP. The acidic TME (pH 6.4–7.0) enables preS1‐PHLIP insertion into the tumor cell membrane. The tumor is then a target of antibody‐mediated cytotoxicity elicited by pre‐existing antiviral humoral immunity, reshaping the tolerogenic TME into an active immune microenvironment.

In this work, we constructed a functional peptide nanomicelle (NM), released in response to the tumor microenvironment (TME), and able to modify the Viral Antigen on the surface of tumor cells to mediate immunotherapy in Head and Neck cancer (**Figure** **1**). The NM structure includes PEG2000 as a hydrophilic component, providing a protective layer that enhances water solubility, prolongs circulation time in vivo, and stabilizes the assembly. The hydrophobic region comprises a matrix metalloproteinase‐2 (MMP2)‐sensitive cleavage site, a preS1_(38–47)_ antigen peptide, and pH (low) insertion peptides (pHLIP). The MMP2‐sensitive segment enables selective activation within the TME, while pHLIP facilitates the anchoring of preS1 epitopes onto tumor cell membranes under acidic conditions typical of the TME. This design aims to reshape the tumor immune microenvironment by prompting tumor cells to display viral antigens, thereby simulating a “giant virus” that can be targeted by pre‐existing anti‐hepatitis B virus (HBV) antibodies. This interaction is intended to activate antibody‐dependent cellular cytotoxicity, leading to enhanced tumor cell destruction. This strategy presents a novel strategy for dealing with tumors featuring ambiguous therapeutic target profiles.

## 2 Results

### 2.1 Design and Characterization of Engineering MMP2‐Responsive PreS1‐pHLIP Nanomicelles

**Figure 2:**
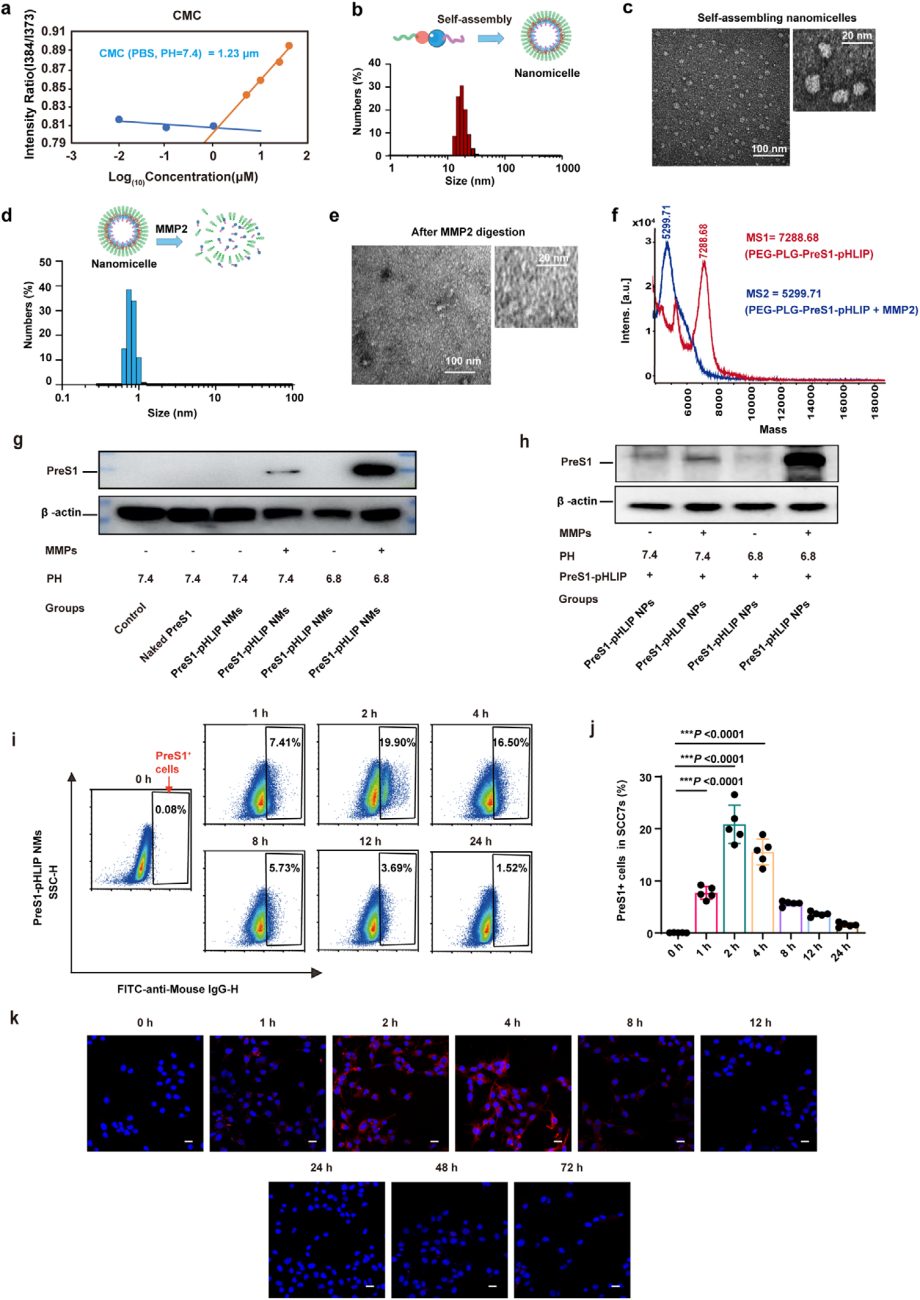
Characterization of self‐assembled NMs and functional evaluation of their response to MMP2 and low PH in vitro. a) CMC curve of preS1‐pHLIP NMs. b) Particle size and c) TEM images of self‐assembled NMs. d) Particle size and e) TEM images of self‐assembled NMs treated with MMP2. f) Molecular weight of PreS1‐pHLIP before and after exposure to MMP2, as determined by TOFMS. g,h) Western blot quantifying membrane preS1 in SCC7 at pH = 7.4 or pH = 6.8, with or without MMP2 treatment. SCC7 cells were incubated with PBS (control), naked preS1 peptide, or preS1‐pHLIP NMs treated with MMP2 or not for 2 h at pH 7.4 or pH 6.8. Membrane preS1‐pHLIP was detected using anti‐preS1 antibodies (g) or serum from mice pre‐immunized with preS1_(38‐47)_ (h). i) Flow cytometry plots showing the proportion of SCC7 cells positive for preS1 after 1, 2, 4, 8, 12, and 24 h exposure to preS1‐pHLIP from MMP2‐treated NMs at 37 °C, pH 6.8, and j) corresponding statistical summary (n = 5). k) Immunofluorescence analysis of PreS1 distribution (red) in SCC7 cells exposed for 1, 2, 4, 8, 12, 24, 48, and 72 h to preS1‐pHLIP from MMP2‐pretreated NMs, by microscopy; scale bar, 20 µm; nuclear counterstain with Hoechst 33 342 (Blue). Statistical significance (*P* value) was calculated using one‐way ANOVA with a Tukey post‐hoc test. ***p < 0.001.

The PreS1 protein, a critical component of the hepatitis B virus surface antigen (HBsAg), has been identified to harbor a high‐affinity binding site for anti‐HBsAg antibodies within its amino acid residues 38–47 (SNNPDWDFNP).^[^ ^7^ ^]^ In this study, we aimed to precisely engineer the PreS1_(38‐47)_ peptide segment into tumor cells, camouflaging them as HBV particles to enable recognition and clearance by pre‐existing hepatitis B antibodies. Guided by the principles of amphiphilic peptide design, we developed a functionalized construct, PreS1‐pHLIP, with its detailed architecture depicted in Figure 1a. The hydrophilic segment is composed of PEG2000, while the hydrophobic region integrates a matrix metalloproteinase‐2 (MMP2)‐sensitive cleavage site, the PreS1_(38–47)_ antigenic peptide, and pH (low) insertion peptides (pHLIP). Additionally, a scrambled peptide sequence lacking the structured PreS1_(38‐47)_ epitope, termed Random‐pHLIP, was synthesized as a control. The critical micelle concentration (CMC) of PreS1‐pHLIP was determined to be 1.23 µM in phosphate‐buffered saline (PBS, pH 7.4) (**Figure** **2**a). Dynamic light scattering (DLS) and transmission electron microscopy (TEM) analyses revealed that PreS1‐pHLIP self‐assembled into uniform nanoscale micelles with an average diameter of ≈18.29 nm at a concentration of 10 µM in PBS (pH 7.4) (Figure 2b‐c and Figure S1, Supporting Information). Subsequently, we investigated the micelle dissociation and PreS1‐pHLIP release triggered by MMP2. DLS and TEM analyses indicated that after 4 h of MMP2 treatment, the micellar morphology was disrupted, confirming successful micelle disassembly (Figure 2d,e). Mass spectrometry further validated the efficient cleavage of PEG2000 by MMP2, releasing the PreS1 peptide (Figure 2f). These findings demonstrate that the MMP2‐responsive self‐assembling peptide system, PreS1‐pHLIP, enables precise control over antigen release.

### 2.2 Acidic Environment Triggers pHLIP‐Mediated PreS1 Peptide Insertion into Tumor Cell Membranes

TME, including that of head and neck squamous cell carcinoma (HNSCC), is typically weakly acidic, with a pH range of 6.4 to 7.0.^[^ ^7^ ^]^ pHLIPs represent a class of peptide molecules that undergo structural and functional changes in response to environmental pH alterations, enabling their insertion into cell membranes under acidic conditions.^[^ ^8^ ^]^ To evaluate the insertion efficiency of preS1‐pHLIP into tumor cell membranes under acidic conditions, we treated PreS1‐pHLIP nanomicelles with MMP2 and assessed membrane anchoring in the SCC7 mouse squamous cell carcinoma cell line. Western blotting analysis revealed a significant enhancement of preS1‐pHLIP insertion at pH 6.8 (simulating the acidic TME) compared to pH 7.4, following MMP2 treatment. And the results using antibodies and serum antibodies demonstrate that the insertion of membrane proteins at pH 6.8 is significantly increased compared to that at pH 7.4, by ≈4.57‐fold and 3.14‐fold, respectively (Figure 2g,h; band intensity data are shown in Figure S2, Supporting Information). To monitor the temporal kinetics of preS1‐pHLIP insertion, MMP2‐treated nanomicelles were incubated with SCC7 cells for various time points (0, 1, 2, 4, 8, 12, and 24 h). Flow cytometric analysis using anti‐preS1 antibodies demonstrated that preS1‐pHLIP insertion peaked at 2 h, with ≈19.8% of cells displaying membrane‐bound preS1‐pHLIP. This was followed by a gradual decline in preS1‐positive cells over time, likely due to endocytosis or degradation. By 24 h, preS1‐pHLIP was nearly undetectable on the cell surface (Figure 2i,j). Fluorescence microscopy corroborated these results, showing significant preS1 enrichment on the tumor cell membrane after 2 and 4 h of incubation (Figure 2k). Collectively, these in vitro findings demonstrate that MMP2‐treated nanomicelles effectively facilitate the rapid and efficient insertion of preS1‐pHLIP into the membranes of HNSCC cells under weakly acidic conditions.

### 2.3 Efficient Tumor Enrichment and Antigen Insertion of PreS1‐pHLIP NMs in Response to MMP2 In Vivo

**Figure 3:**
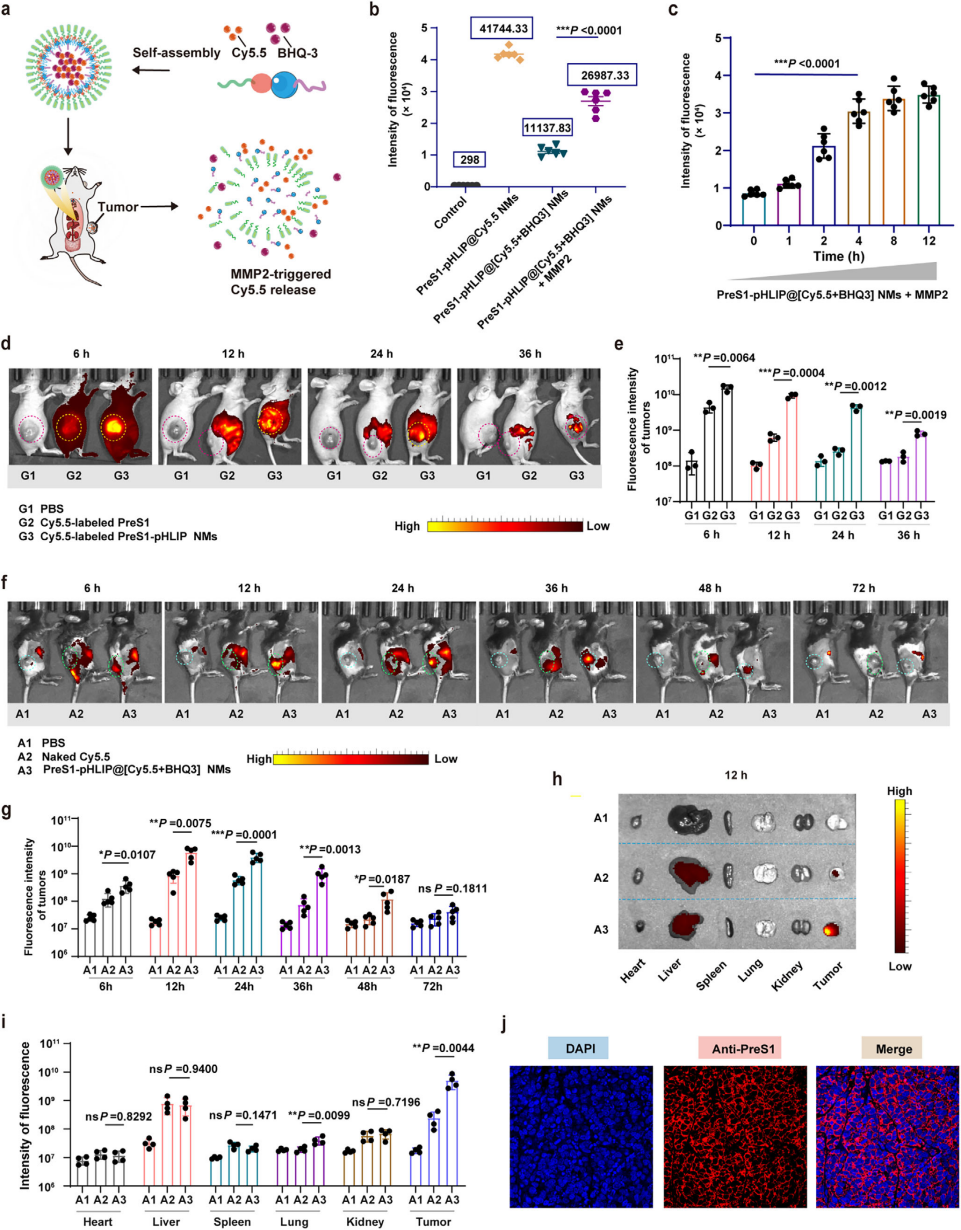
Assessment of preS1‐pHLIP NM intratumoral release in vivo for tumor targeting. a) Graphic representation of the “quench‐recovery” strategy used to monitor NMs release within tumors. b) In vitro pre‐validation of the quench‐recovery strategy with preS1‐pHLIP@[Cy5.5+BHQ3] NMs (preS1‐pHLIP@[Cy5.5+BHQ3] NMs + MMP2) and controls (PBS only {Control}, no quencher {preS1‐pHLIP@Cy5.5 NMs}, and untreated {preS1‐pHLIP@[Cy5.5+BHQ3]}), which were incubated or not with MMP2 (2 µg mL^−1^) at a 1:1 volume ratio for 4 h. Cy5.5 fluorescence was read using a spectrophotometer (n = 6). c) Fluorescence kinetics from preS1‐pHLIP@[Cy5.5+BHQ3] NMs treated with MMP2 for 0, 1, 2, 4, 8 and 12 h, measured in a spectrophotometer (n = 6). d) Representative images and e) statistical summary of the fluorescence kinetics in subcutaneous SCC7 tumor‐bearing mice injected intravenously with PBS (group 1, G1), free Cy5.5 (group 2, G2), or Cy5.5‐labeled preS1‐pHLIP NMs (group 3, G3), and measured at 6, 12, 24, and 36 h post‐injection (n = 3 mice/group). f) Representative images and g) statistical summary of the fluorescence kinetics in subcutaneous SCC7 tumor‐bearing mice injected intravenously with PBS (group 1, A1), free Cy5.5 (group 2, A2), or preS1‐pHLIP@[Cy5.5+BHQ3] NMs (group 3, A3), and measured at 6, 12, 24, 36, 48, and 72 h post‐injection (n = 5 mice/group). h) Representative images of major organs and tumor tissues, and i) statistical summary of the fluorescence kinetics from subcutaneous SCC7 tumor‐bearing mice injected intravenously with PBS (group 1, A1), free Cy5.5 (group 2, A2), or preS1‐pHLIP@[Cy5.5+BHQ3] NMs (group 3, A3), and sacrificed at 12 h post‐injection (n = 4 mice/group). j) Representative immunofluorescence images of preS1^+^ cells in tumor tissues, 12 h after preS1‐pHLIP NMs injection (n = 4). Statistical significance (*p* value) was calculated using one‐way ANOVA with a Tukey post‐hoc test. *p < 0.05; **p < 0.01; ***p < 0.001.

To validate the tumor‐specific accumulation of PreS1‐pHLIP NMs in response to MMP2, we developed a fluorescence probe encapsulating Cyanine 5.5 (Cy5.5) and the BHQ_3_ quencher within the nanomaterials to monitor MMP2‐triggered disassembly in vivo (**Figure** **3**a). First, we assessed the fluorescence quenching efficiency of the probe in vitro. Compared to Cy5.5@PreS1‐pHLIP NMs, the [Cy5.5+BHQ_3_] @PreS1‐pHLIP NMs exhibited a fluorescence quenching rate of 73.8% due to BHQ3 proximity (Figure 3b). Upon incubation with MMP2 for 2 h, the quenching rate decreased to 51.78%, indicating fluorescence recovery. Time‐dependent fluorescence intensity changes were further monitored for 1, 2, 4, 8, and 12 h, with the fluorescence recovery at 12 h defined as 100%. After 2 and 4 h of MMP2 treatment, the dissociation rates of [Cy5.5+BHQ_3_] @PreS1‐pHLIP NMs were 50.74% and 88.15%, respectively (Figure 3c), confirming the successful construction of the MMP2‐responsive fluorescence probe.

The biodistribution of PreS1‐pHLIP NMs was subsequently evaluated in vivo. NHS‐Cy5.5 is utilized to label NMs as a fluorescent imaging probe, and NHS‐Cy5.5 labeling does not interfere with the assembly of NMs or their responsive disintegration to MMP2 (Figure S3, Supporting Information). Compared to free Cy5.5‐PreS1, Cy5.5@PreS1‐pHLIP NMs exhibited significantly higher fluorescence signals at the tumor site at 6 and 12 h post‐injection. Notably, at 24 and 36 h, fluorescence retention at the tumor site remained more pronounced with Cy5.5@PreS1‐pHLIP NMs compared to free Cy5.5‐PreS1 peptide (Figure 3d,e), which may be attributed to the enhanced permeability and retention effect and prolonged systemic circulation conferred by PEGylation.^[^ ^10^ ^]^ These results demonstrated the efficient tumor‐targeting capability of PreS1‐pHLIP NMs.

We further monitored the MMP2‐responsive behavior of PreS1‐pHLIP NMs in the MMP2‐enriched tumor microenvironment (TME) using the [Cy5.5+BHQ_3_] @PreS1‐pHLIP NMs fluorescence probe. As shown in Figure 3f, the fluorescence release was localized predominantly at the tumor site, with the peak fluorescence intensity observed at 12 h post‐injection (Figure 3f,g, Figure S4, Supporting Information). Ex vivo imaging of major organs and tumor tissues confirmed significant fluorescence release and accumulation specifically in the tumor at 12 h (Figure 3h,i, Figure S4, Supporting Information). Immunofluorescence analysis of tumor sections further revealed that PreS1‐pHLIP insertion into tumor cell membranes peaked at 12 h post‐injection (Figure 3j and Figure S5, Supporting Information).

In summary, these findings demonstrate that PreS1‐pHLIP NMs effectively accumulate at tumor sites and respond to the MMP2‐rich TME, releasing PreS1‐pHLIP and mediating its targeted insertion into tumor cell membranes via pHLIP. This represents a critical step in reprogramming antiviral immunity toward antitumor immune responses.

### 2.4 PreS1‐pHLIP NMs Utilize Pre‐existing Immunity to Facilitate Antitumor Therapy and Tumor Microenvironment Remodeling

**Figure 4:**
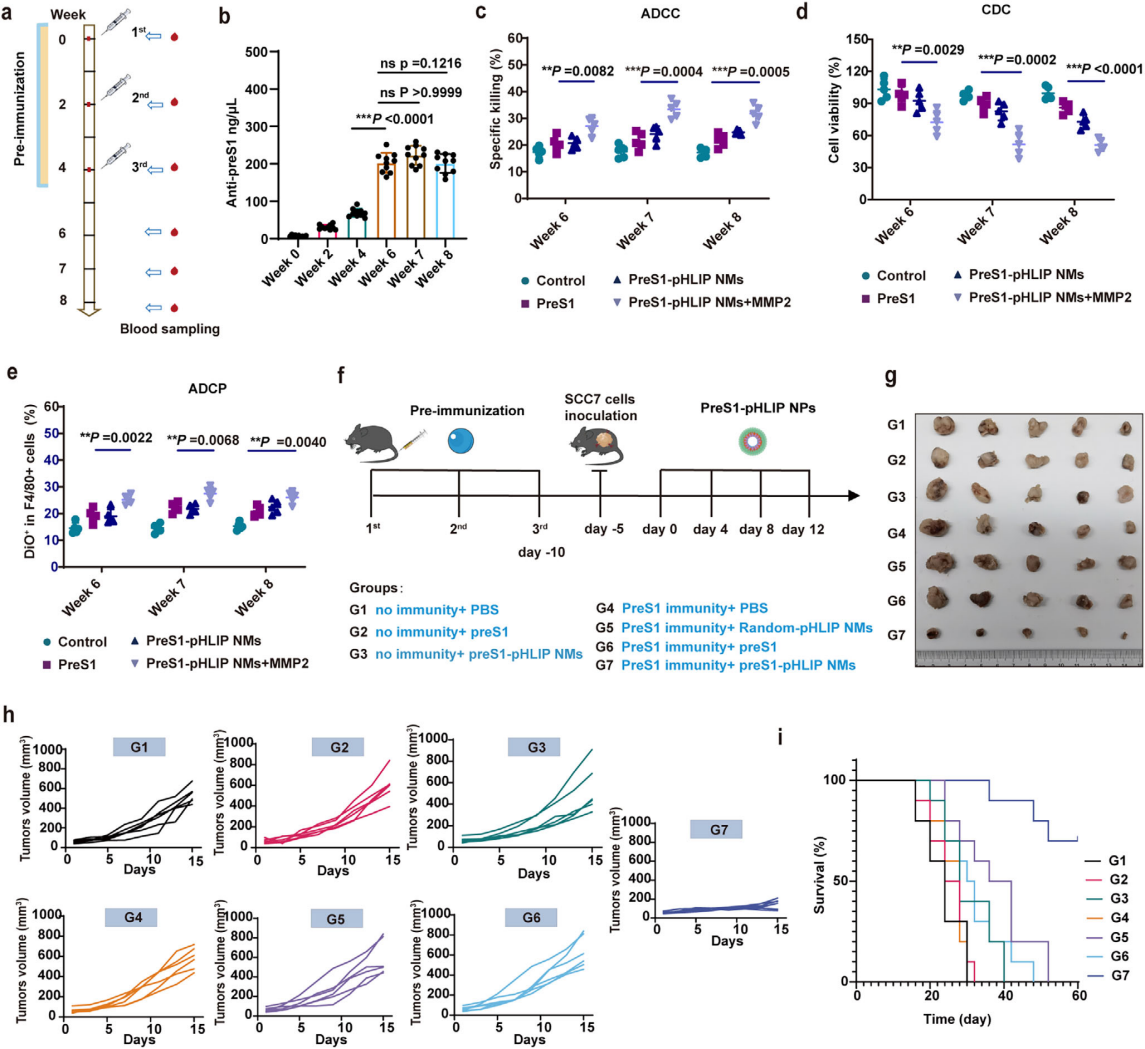
In vitro and in vivo evaluation of anti‐preS1 antibody/preS1‐pHLIP NMs‐mediated antitumor effect. a) Graphic representation of the preimmunization schedule with preS1 peptide. The red drop symbols indicate blood sampling and serum collection. b) PreS1_(37‐48)_‐specific IgG concentration in serum, measured by ELISA at week 0, 2, 4, 6, 7, and 8 after prime immunization (n = 10). c–e) PreS1_(37‐48_) antibody‐mediated SCC7 killing assessed in vitro by (c) ADCC, using lymphokine‐activated killer cells, (d) CDC, with complement and sera from immunized mice, and (e) ADCP, using BMDMs; SCC7 cells were pretreated as indicated; “Control” corresponds to PBS pretreatment, (n = 5). f) Graphic representation of the preimmunization and treatment schedule used to assess anti‐preS1 antibody/preS1‐pHLIP NMs‐mediated antitumor in vivo. C3H mice were vaccinated or not with three doses of preS1 peptide. 5 days after the last immunization, the mice were inoculated SCC7 cells subcutaneously. After five days, the tumor‐bearing mice were treated every 4 days for a total of four times. The first treatment was recorded as day 0, the tumor size was measures every other day, and the mice were sacrificed for immune analysis on day 20. g) Pictures showing tumors isolated on day 20. h) Growth curves of the tumors in individual mice (n = 6). i) Kaplan‐Meier survival analysis (n = 10). Statistical significance (*p* value) was calculated using one‐way ANOVA with a Tukey post‐hoc test. *p < 0.05; **p < 0.01; ***p < 0.001.

To evaluate the antitumor efficacy of PreS1‐pHLIP NMs, we established a pre‐existing anti‐PreS1 antibody model to simulate individuals vaccinated against HBV. Briefly, immunocompetent C3H mice were immunized with PreS1 polypeptide and Alum adjuvant (**Figure** **4**a). 2 weeks after the last injection (week 6), serum anti‐PreS1 antibody titers, as measured by enzyme‐linked immunosorbent assay (ELISA), had a mean value of 202.89 ng µL^−1^, which remained stable through week 7 and 8. (Figure 4b). The antitumor efficacy of anti‐PreS1 antibodies was then evaluated in vitro. In the antibody‐dependent cellular cytotoxicity (ADCC) assay, SCC7 cells treated with PBS, PreS1, PreS1‐pHLIP NMs, or PreS1‐pHLIP NMs+MMP2 served as targets, and primary natural killer (NK) cells were used as effectors. Lactate dehydrogenase (LDH) release confirmed that SCC7 cells pre‐treated with PreS1‐pHLIP NMs+MMP2 elicited significantly stronger ADCC compared to other treatments (Figure 4c). Complement‐dependent cytotoxicity (CDC) assays similarly demonstrated that SCC7 cells treated with PreS1‐pHLIP NMs+MMP2 exhibited the lowest viability upon incubation with complement and immune sera (Figure 4d). For antibody‐dependent cellular phagocytosis (ADCP), SCC7 cells were fluorescently labeled with DIO and co‐cultured with bone marrow‐derived macrophages (BMDMs) and sera from preS1‐preimmunized mice for 6 h. Flow cytometry showed the highest phagocytic activity in PreS1‐pHLIP NMs+MMP2‐treated cells (Figure 4e). These results collectively demonstrated that anti‐preS1 antibodies bound to the surface‐inserted PreS1 antigen on SCC7 cells by PreS1‐pHLIP NMs, facilitating tumor cell killing through ADCC, CDC, and ADCP pathways.

To confirm the in vivo antitumor efficacy of PreS1‐pHLIP NMs via mobilization of anti‐preS1 immunity, C3H mice with or without pre‐existing anti‐PreS1 antibody were subcutaneously inoculated with SCC7 cells. Mice were intravenously injected with indicated formulations at 4‐day intervals for five doses (Figure 4f). All mice exhibited normal weight throughout treatment (Figure S6, Supporting Information). While mice without pre‐existing anti‐PreS1 antibody displayed no significant antitumor effects, PreS1‐pHLIP NMs‐treated mice with pre‐existing anti‐PreS1 antibody demonstrated substantial tumor suppression (Figure 4g,h). Remarkably, 70% of mice with pre‐existing anti‐PreS1 antibody in the PreS1‐pHLIP NMs‐treated group survived beyond 60 days, whereas no mice from other groups survived (Figure 4i). These findings highlight the potent antitumor activity of PreS1‐pHLIP NMs through mobilization of pre‐existing anti‐preS1 immunity, supporting the concept of repurposing antiviral immunity for antitumor responses via intratumoral antigen release and transmembrane insertion.

**Figure 5:**
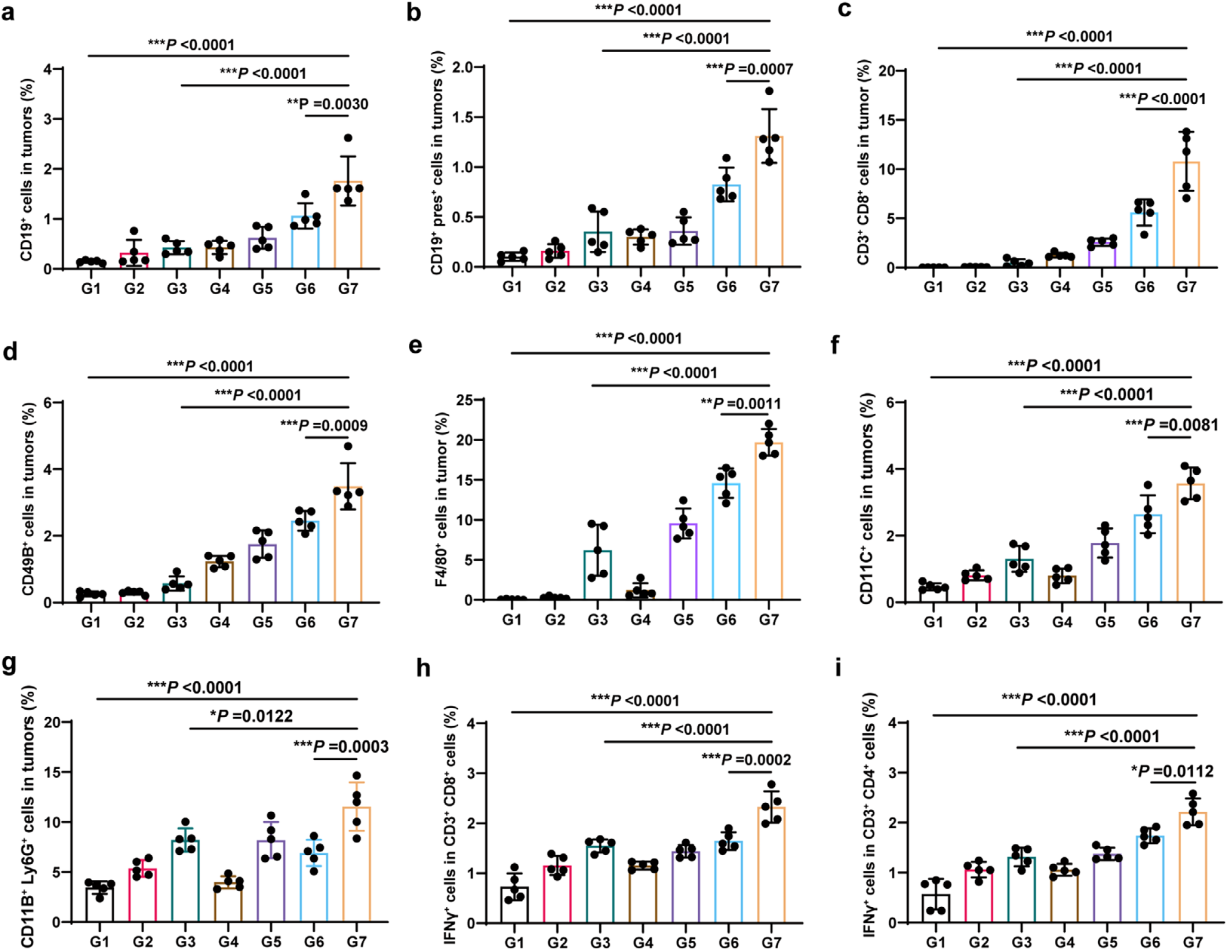
TME remodeling and activation of T cell adaptive responses induced by PreS1‐pHLIP NMs in HNSCC. The data were collected using the experimental setup shown in Figure 4f. Immune cell infiltrates in tumors and tumor antigen‐specific T cells in spleen were analyzed by flow cytometry on day 20 after the beginning of treatment. Proportions of a) CD19^+^, b) preS1^+^CD19^+^ B cells, c) CD3^+^CD8^+^ T cells, d) CD49B^+^ NK cells, e) F4/80^+^ macrophages, f) CD11C^+^ DC cells, and g) CD11B^+^Ly6G^+^ neutrophils in tumor tissues (n = 5). h,i) Proportion of IFN‐γ^+^ cells among splenic h) CD3^+^CD8^+^ T cells, and i) CD3^+^CD4^+^ T cells after ex vivo re‐stimulation with tumor antigens (n = 5). Statistical significance (*p* value) was calculated using one‐way ANOVA with a Tukey post‐hoc test. *p < 0.05; **p < 0.01; ***p < 0.001.

To elucidate the effects of PreS1‐pHLIP NMs on the tumor immune microenvironment (TME), flow cytometry was performed to characterize intratumoral immune cell populations. PreS1‐pHLIP NMs treatment in mice with pre‐existing anti‐PreS1 antibody led to significant infiltration of CD19^+^ and preS1+CD19^+^ B cells into the tumor (**Figure** **5**a,b). Given the central role of B cell memory in antiviral protection,^[^ ^11^ ^]^ increased antigen‐specific B cells suggest enhanced antiviral immunity within the TME. In addition to B cell recruitment, PreS1‐pHLIP NMs significantly increased intratumoral CD3^+^, CD3^+^CD4^+^, and CD3^+^CD8^+^ T cell populations (Figure 5c,d, Figure S7, Supporting Information), indicating the mobilization of T cell‐mediated immunity for tumor elimination. This adaptive immune response was accompanied by increased proportions of innate immune cells, including CD49^+^ NK cells, CD11C^+^ dendritic cells (DCs), and CD11b^+^Ly6G^+^ neutrophils (Figure 5e–g). Furthermore, PreS1‐pHLIP NMs treatment recruited significant numbers of F4/80^+^CD86^+^ M1‐polarized macrophages and mature DC cells (CD80^+^CD86^+^ cells in CD11C^+^ cells), and significantly decreased F4/80^+^CD206^+^ M2‐polarized macrophages, CD11B^+^GR1^+^ MDSCs cells and CD4^+^Foxp3^+^ Tregs cells into the tumor tissue (Figure S8, Supporting Information). Molecular analysis of chemokines within tumors revealed elevated expression of CCL2, CCL7, CCL9, CCL24, CXCL1, CXCL2, CXCL5, CXCL10, and CXCL11 following PreS1‐pHLIP NMs treatment (Figure S9, Supporting Information), potentially explaining the observed immune cell infiltration. Furthermore, tumor antigen release during immune‐mediated tumor cell killing may lead to antigen presentation by antigen‐presenting cells (APCs), thereby activating antigen‐specific T cell responses. Splenocytes from treated mice were re‐stimulated with tumor lysate, and flow cytometry confirmed the activation of tumor antigen‐specific T lymphocytes in the PreS1‐pHLIP NMs‐treated immunized group (Figure 5h–i).

**Figure 6:**
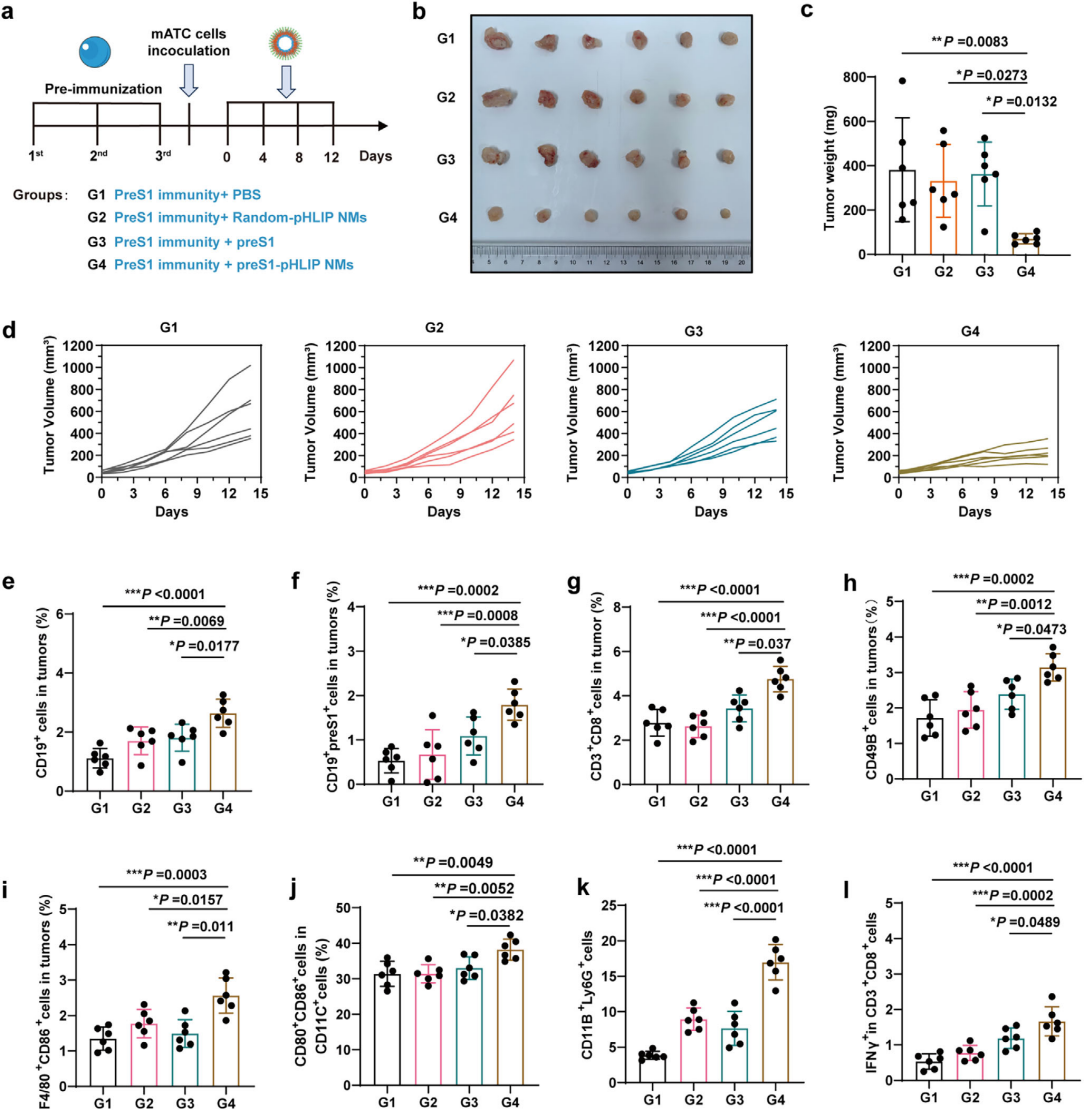
Antitumor effect and TME remodeling induced by PreS1‐pHLIP NMs in ATC. a) Schematic illustration of the experiment schedule and group identification/key. 6‐week‐old C57 mice were pre‐immunized by intramuscular injection of preS1 peptide three times at 2‐week intervals, and then were randomly divided into 4 groups as follows: group 1 (G1), pre‐immunity + PBS, group 2 (G2), pre‐immunity + Random NMs, group 3 (G3), pre‐immunity + preS1, group 4 (G4), pre‐immunity + preS1‐pHLIP NMs. 5 days after the last immunization, mATCs cells (2 × 10^6^ cells each) were inoculated subcutaneously, and treatment was initiated when the tumor had grown to 50 mm^3^. The first injection was recorded as day 0, and treatments were performed every 4 days. Tumor volumes were measured every other day until day 14. On day 16, the mice were euthanized for tumor photography, weighing, and immune analysis. b) Tumor image, c) tumor weight, and d) growth curves of subcutaneous tumors in each mouse. (n = 5). Proportions of e) CD19^+^ and f) preS1^+^CD19^+^ B cells, g) CD3^+^CD8^+^ T cells, h) CD49B^+^ NK cells, i) F4/80^+^CD86^+^ M1‐polarized macrophages, j) CD80^+^CD86^+^ DC cells and k) CD11B^+^Ly6G^+^ neutrophils in tumor tissues (n = 5). l) Proportions of IFN‐γ^+^ cells in CD3^+^CD8^+^ T cells in tumor tissues stimulated with the cell lysate. (n = 5). The data were processed using GraphPad Prism 8 and are presented as the mean ± SD. The *p* values were determined using one‐way ANOVA with a Tukey post‐hoc test. *p < 0.05; **p < 0.01; ***p < 0.001.

Anaplastic thyroid cancer (ATC) is one of the most aggressive and lethal malignancies among head and neck tumors.^[^ ^9^ ^]^ To further explore the applicability of PreS1‐pHLIP NMs for the treatment of head and neck tumors, we chosen the ATC model to antitumor efficacy evaluation and immunological analyses. C57 mice with pre‐existing anti‐PreS1 antibody were subcutaneously inoculated with mATC cells. Mice were intravenously injected with indicated formulations at 4‐day intervals for five doses (**Figure** **6**a). Treatment with Random‐pHLIP NMs or preS1 peptide did not elicit marked antitumor activity, while the preS1‐pHLIP NMs showed a remarkable antitumor effect (Figure 6b–d). Subsequently, we also further assessed the infiltration of immune cells in the TEM using flow cytometry. Compared with Random‐pHLIP NMs and preS1 peptide treatment, preS1‐pHLIP NMs treatments increased the proportions of CD19^+^ and preS1^+^CD19^+^ B cells (Figure 6e,f). Additionally, preS1‐pHLIP NMs treatment recruited significant numbers of CD3^+^CD8^+^ T cells, CD49B^+^ NK cells, F4/80^+^CD86^+^ M1‐polarized macrophages, CD80^+^CD86^+^ DC cells and CD11B^+^Ly6G^+^ neutrophils into the tumor tissue (Figure 6g–j). And preS1‐pHLIP NMs obviously activated antigen‐specific T lymphocytes in splenocytes (Figure 6k). Collectively, the locally released preS1‐pHLIP NMs altered the promoted TME remodeling via recruitment of various immune cells.

In conclusion, PreS1‐pHLIP NMs facilitate intratumoral release and transmembrane insertion of viral antigen, effectively mobilizing pre‐existing anti‐preS1 memory immunity. This strategy not only reshapes the TME by recruiting diverse immune cell subsets but also induces tumor antigen‐specific adaptive immunity, enabling robust antitumor responses.

### 2.5 PreS1‐pHLIP NMs Mobilize Antiviral Immunity to Suppress Postoperative Recurrence and Metastasis in HNSCC

**Figure 7:**
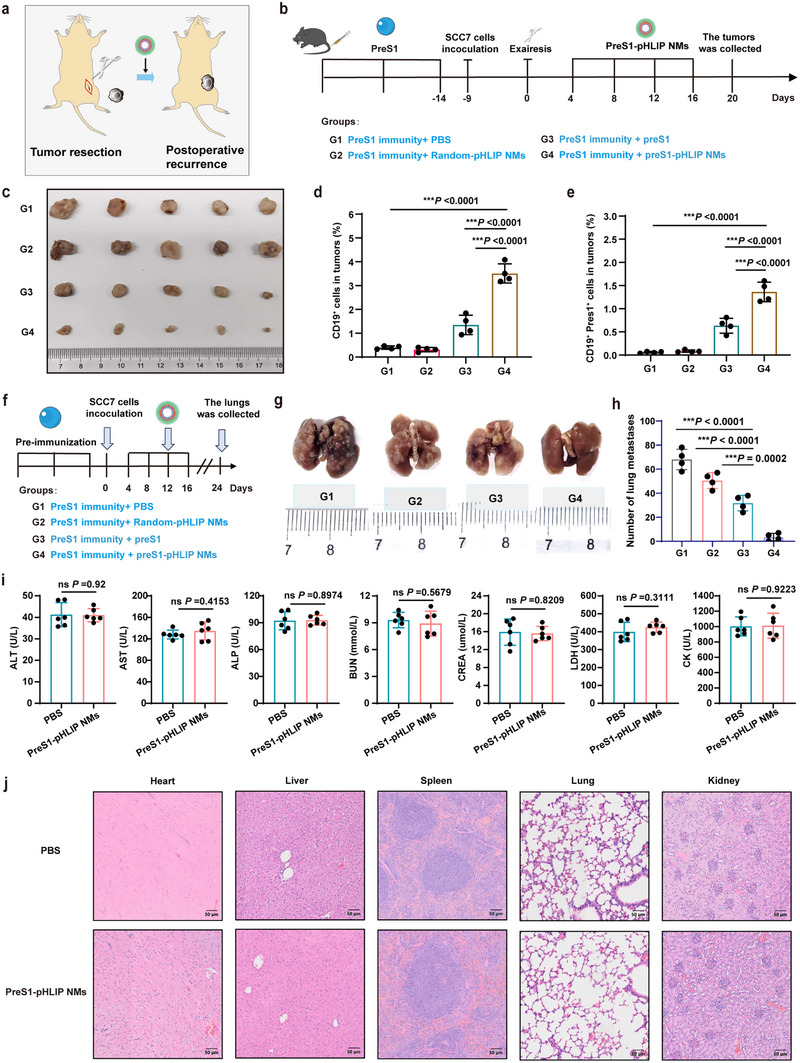
Evaluation of preS1‐pHLIP NMs effect in postoperative recurrent and metastatic HNSCC, and biosafety analysis. a,b) Graphic representation of the protocol used to establish postoperative recurrent HNSCC. Mice preimmunized with preS1 peptide were inoculated with SCC7 tumor cells subcutaneously. When the tumor volume reached ≈200 mm^3^ (about 9 days post inoculation), the tumor was surgically removed. The mice were then treated with different drugs on days 4, 8, 12, and 16 post‐surgery, and sacrificed at days 20 post surgery; group 1(G1), PBS; group 2 (G2), Random‐pHLIP NMs; group 3 (G3), free preS1; group 4 (G4), PreS1‐pHLIP NMs. c) Pictures of tumors isolated at experimental endpoint (n = 5). d,e) Flow cytometric analysis of the proportions of CD19^+^ (d) and preS1^+^CD19^+^(e) B cells in tumor tissues (n = 4). f) Graphic representation of the protocol used to establish metastatic HNSCC. PreS1‐preimmunized mice were inoculated SCC7 cells intravenously. The mice were treated with different drugs on days 4, 8, 12, and 16 post inoculation. g) Lungs pictures and h) numbers of metastatic nodules (n = 4). i,j) Biosafety assessment in vivo; i) blood biochemical analysis and j) histological evaluation of major organs collected after four doses of intravenous preS1‐pHLIP NMs and stained with H&E, (n = 6). Statistical significance (*p* value) was calculated using one‐way ANOVA with a Tukey post‐hoc test (d,e, h), or unpaired *t*‐test (i). *p < 0.05; **p < 0.01; ***p < 0.001; ns, no significant difference.

To determine the suitability of mobilizing antiviral immunity for suppressing postoperative recurrence and metastasis, we established both a postoperative resection and recurrence model and a lung metastasis model of HNSCC. In the postoperative recurrence model, preS1‐preimmunized C3H mice were subcutaneously inoculated with SCC7 cells. Once tumors reached ≈200 mm^3^, they were surgically resected, leaving residual microscopic tumor tissue to simulate recurrence, followed by wound closure (**Figure** **7**a). After a 4‐day recovery period, when tumors had regrown to ≈50 mm^3^, mice were treated with PreS1‐pHLIP NMs, free PreS1 peptide, Random‐pHLIP NMs, or PBS as control (Figure 7b). Following five doses of treatment, PreS1‐pHLIP NMs significantly delayed tumor regrowth, whereas control groups exhibited rapid recurrence (Figure 7c and Figure S10, Supporting Information). The enhanced antitumor effect was associated with increased infiltration of CD19⁺ and preS1⁺CD19⁺ B cells in tumor tissues (Figure 7d,e), indicating a robust immune response specifically induced by PreS1‐pHLIP NMs. These results highlight the therapeutic potential of PreS1‐pHLIP NMs for managing postoperative recurrent HNSCC.

For the lung metastasis model, SCC7 cells were intravenously injected into preS1‐preimmunized mice to induce pulmonary metastases (Figure 7f). 4 days post‐injection, mice were treated with four doses of PreS1‐pHLIP NMs, free PreS1 peptide, Random‐pHLIP NMs, or PBS at 4‐day intervals. 20 days after treatment initiation, lung tissue analysis revealed an ≈80% reduction in metastatic nodules in mice treated with PreS1‐pHLIP NMs compared to controls (Figure 7g,h). These findings demonstrate the efficacy of the PreS1‐pHLIP NMs system in targeting metastatic HNSCC and highlight its potential utility as a tumor vaccine platform.

To assess the systemic toxicity of intravenous PreS1‐pHLIP NMs, mice received five doses of the nanomaterial, each containing 25 µM of PreS1 peptide. Blood biochemical analyses revealed no significant alterations in alanine transaminase (ALT), aspartate transaminase (AST), alkaline phosphatase (ALP), blood urea nitrogen (BUN), creatinine (CREA), LDH, or creatine kinase (CK) levels (Figure 7i). Additionally, histopathological examination of major organs, including the heart, liver, spleen, lungs, and kidneys, showed no noticeable abnormalities upon hematoxylin and eosin (H&E) staining (Figure 7j). In summary, PreS1‐pHLIP NMs demonstrated remarkable antitumor efficacy in models of postoperative recurrence and lung metastasis without inducing systemic toxicity. These findings underscore the potential of PreS1‐pHLIP NMs as a novel, safe, and effective strategy for immunotherapy of recurrent and metastatic HNSCC.

## 3 Conclusion 

Abundant antigen‐specific antibodies and antigen‐specific B cells produced after virus infection or antiviral vaccination.^[^ ^6a^ ^]^ In this study, we developed and validated a novel nanoplatform, PreS1‐pHLIP NMs, that harnesses pre‐existing antiviral immunity to overcome the challenges of tumor heterogeneity and therapeutic target ambiguity in head and neck cancer. By facilitating the targeted insertion of viral antigens onto tumor cell membranes within the acidic tumor microenvironment, PreS1‐pHLIP NMs effectively mobilized pre‐existing anti‐preS1 immune responses to induce robust antibody‐mediated tumor cell killing through ADCC, CDC, and ADCP pathways.

In preclinical models, PreS1‐pHLIP NMs demonstrated significant antitumor efficacy in postoperative recurrence and lung metastasis models of HNSCC, as well as in ATC, with treated mice exhibiting delayed tumor regrowth, reduced metastatic burden, and prolonged survival. Notably, the platform's ability to remodel the tumor immune microenvironment—recruiting preS1‐specific B cells, adaptive T cells, and innate immune subsets—was consistent across both HNSCC and ATC models, despite their divergent pathophysiologies. Importantly, this therapeutic effect was accompanied by profound remodeling of the tumor immune microenvironment, characterized by the recruitment of preS1‐specific B cells, enhanced infiltration of adaptive immune subsets (CD3⁺, CD4⁺, and CD8⁺ T cells), and activation of innate immune cells (NK cells, DCs, and neutrophils). Moreover, PreS1‐pHLIP NMs treatment led to elevated chemokine expression, further supporting immune cell recruitment and activation within the tumor milieu. Systemic toxicity evaluations revealed that PreS1‐pHLIP NMs are well tolerated, with no significant changes in biochemical markers or histopathological abnormalities observed in major organs, underscoring the safety profile of this approach.

Collectively, our findings demonstrate that PreS1‐pHLIP NMs represent an innovative and effective strategy to repurpose antiviral immunity for cancer immunotherapy, achieving targeted tumor killing and immune microenvironment remodeling without systemic toxicity. The broad efficacy observed in both HNSCC and ATC models underscores the versatility of this approach for diverse head and neck malignancies. This strategy holds significant promise for the treatment of tumors with unclear targets and complex target selection processes. Additionally, it may broaden the scope of drug design for tumor vaccines, antibody‐drug conjugates, and immune checkpoint blockade therapies. Future studies will focus on enhancing the durability of antitumor immunity, as suggested by the long‐term follow‐up data in Figure S20, Supporting Information. Certainly, the current nanosystem still has certain limitations, such as limited tumor‐killing capability. Strategies such as enhancing in vivo half‐life, or combining it with traditional therapies including surgery, chemotherapy, and immunotherapy, may be promising approaches to achieve more efficient treatment outcomes.

## 4 Experimental Section

### Reagents and Materials

PEG2000‐PLGLAG (MMP2 restriction site)‐preS1_(38‐47)_‐pHLIP, PEG2000‐PLGLAG (MMP2 restriction site)‐Random (RGVPHIVMVD)‐pHLIP and preS1_(38‐47)_ peptide were constructed using solid phase peptide synthesis at China Peptides Co., Ltd. (Shanghai, China). Recombinant MMP‐2 Protein (Sino Biological, China, Cat: 10082‐HNAH) was purchased from Sino Biological Co., Ltd. China. P‐aminophenyl‐mercuric acetate (APMA, GENMED, USA, Cat: GMS12197.2), Collagenase D (Sigma, USA, Cat: 11 088 882 001) and DNase I (Solarbio, China, Cat: D8071) were purchased from LABLEAD Inc (Hangzhou, China).

The antibodies used for flow cytometry were as follows: anti‐mouse CD19 APC (BioLegend, USA, Cat: 115 512), anti‐mouse CD3 APC (BioLegend, USA, Cat: 100 312), anti‐mouse CD11C APC (BioLegend, Cat: 117 310), anti‐mouse CD8 FITC (Biolegend, Cat: 100 706), anti‐mouse CD49B FITC (Biolegend, Cat: 103 503), anti‐mouse Ly6G FITC (Biolegend, Cat: 127 606), anti‐mouse CD4 PE (Invitrogen, USA, Cat: 12‐0042‐85), anti‐mouse CD11B PE (Biolegend, Cat: 101 208), anti‐mouse F4/80 PE/Cyanine7 (Biolegend, Cat: 123 114), anti‐mouse IFNγ PE/Cyanine7 (Biolegend, Cat: 505 826), anti‐mouse CD86 PE/Cyanine7 (Biolegend, Cat: #105 014), anti‐mouse Ly‐6G/Ly‐6C (Gr‐1) FITC (BioLegend, Cat: 108 406), anti‐mouse/human CD11b PerCP/Cyanine5.5 (BioLegend, Cat: 101 228), anti‐mouse CD80 APC (BioLegend, Cat: 104 714), anti‐mouse CD80 Brilliant Violet 605 (BioLegend, Cat: 104 729), anti‐mouse CD206 PE (Biolegend, Cat: 141 706), anti‐mouse CD4 FITC (Biolegend, Cat: 100 406), anti‐Mouse F4/80 eFluor 450 (Thermo Fisher Scientific, USA, Cat: 48‐4801‐82), anti‐Mouse Foxp3 eFluor 450 (Thermo Fisher Scientific, Cat: 48‐5773‐82). The antibodies used for western blots and Immunofluorescence staining were as follows: anti‐mouse preS1 antibody (Santa Cruz, USA, Cat: sc‐57761), FITC‐conjugated goat anti‐mouse IgG (Proteintech, USA, Cat: SA00003‐1), Alexa Fluor 594‐conjugated anti‐mouse IgG (eBioscience, USA, Cat: **A‐11005**), and anti‐ mouse β‐Actin (Santa Cruz, Cat: sc‐517582).

### Cell Culture and Animals

Mouse head and neck squamous cell line SCC7 was purchased from the China Center for Culture Collection. The cells were cultured in RPMI 1640 medium (Biological Industries, Israel, Cat: 01‐100‐1A) containing 10% fetal bovine serum (FBS, Biological Industries, Cat: 04‐001‐1A), 100 units mL^−1^ penicillin and 0.1 mg mL^−1^ streptomycin (Biological Industries, Cat: 03‐031‐1B) under humid atmosphere at 37 °C and 5% CO2.

5‐week‐old BALB/c nude mice and 6‐week‐old C3H mice were purchased from Charles River, Beijing, China. The mice were kept in a specific pathogen‐free environment where artificial light was provided in a 12 h light /dark cycle. Food and water were available ad libitum. This study complied with the relevant ethical norms for animal experiments and research, and all animal protocols were approved by the Animal Care and Use Committee of the National Nanoscience and Technology Center.

### Determination of the CMC of PreS1‐pHLIP Complex Peptide

The CMC of PreS1‐pHLIP complex peptide was measured by fluorescence probe method as described previously.^[^ ^10^ ^]^ The PreS1‐pHLIP complex peptide was ultrasonically dissolved in PBS with a peptide gradient ranging from 0.01 to 100 µM. Pyrene (MedChemExpress, USA, Cat: HY‐103609) were dissolved in citrate buffer (2 mM, PH = 6.2). Pyrene saturated solution (1.22%, m/v) and peptide solutions at different concentrations were thoroughly mixed and incubated for 1 h. The pyrene fluorescence intensity of each solution was determined at the excitation wavelength of 334 nm and the wavelength range of 350–450 nm. The ratio of fluorescence intensity of pyrene at the first peak (370–373 nm, I_1_) to the third peak (381–384 nm, I_3_) was taken as the Y axis, and the Log_10_ value of complex peptide concentration was taken as the X axis for linear fitting. The inflection point corresponding to the change in curve behavior was the CMC value of micelle.

### Preparation and Characterization of preS1‐pHLIP NMs

The CMC was 1.23 µM. Peptide solutions at 1.25, 2.5, 5, and 10 µM were incubated in PBS (PH = 7.0) at room temperature for 2 h, and particle size analysis was performed by DLS (Zetasizer Pro, Malvern Panalytical, UK). To evaluate the morphology of the complex peptide after self‐assembly, 5 µM complex peptide was incubated in PBS for 2 h, and the morphology of the NMs was observed under TEM. To detect the NMs’ response to the dissociation and release of MMP2, MMP2 (100 µg mL^−1^) and APMA (1 mM) were pre‐activated by TCNB buffer (composed of 100 mM Tris, 10 mM CaCl_2_, 150 mM NaCl, and 0.05% Brij‐35, pH = 7.4) at 37 °C for 4 h to obtain an activated MMP2 solution. Then, 0.2 µg mL^−1^ activated MMP‐2 and 5 µM composite peptide self‐assembled NMs were mixed and incubated at 37 °C for 4 h at a 1:1 volume ratio. After enzyme digestion, the size and morphology of the reaction product were analyzed by DLS and TEM. The fragments PreS1‐pHLIP were treated with MMP2, and the digestion product was concentrated and desalted by ultrafiltration. The changes in molecular weight of the fragments before and after digestion were measured by Time‐of‐flight Mass Spectrometry (TOFMS, Autoflex MAX, Bruker, Germany).

To analyze the efficiency of preS1‐pHLIP cell membrane insertion, SCC7 cells were seeded in 24‐well plates. In the meantime, 20 µM preS1‐pHLIP NMs were pretreated with activated MMP2 solution (0.2 µg mL^−1^) for 4 h. The enzyme‐digested NMs were incubated with SCC7 cells for 1, 2, 4, 8, 12 or 24 h at 37 °C, pH 6.8. Then, the cells were collected and washed three times with PBS (pH = 6.8). Anti‐preS1 antibody acts as a primary antibody to label preS1 polypeptide on the cell surface (37 °C, 1 h), and FITC‐conjugated goat anti‐mouse IgG was co‐incubated with primary antibody labeled cells (37 °C, 1 h). Flow cytometry was used to analyze the proportion of preS1 inserted on the surface of tumor cells after different treatment times. In addition, preS1 on the surface of SCC7 cells’ membrane was visualized by immunofluorescence according to the immunofluorescence method described below.

### Fluorescent Probe Preparation and In Vivo Fluorescence Imaging of PreS1‐pHLIP NMs

Fluorescent probe preparation: 0.5 mg preS1‐pHLIP, 0.1 mg Cy5.5 (MedChemExpress, Cat: HY‐D0924) and 0.1 mg BHQ‐3 (New Research Biosience, China, Cat: Y‐R‐3189, China) were dissolved in 10 µL DMSO. Then, the mixed solution was completely dispersed into 1 mL of PBS. After incubation at room temperature for 2 h, the product was purified by a 30‐KDa molecular weight cut‐off ultrafiltration tube (Merck Millipore, UFC5030BK, USA) and fluorescent nanoprobes were obtained.

The response of the nanoprobe was evaluated in vitro: the fluorescent probe preS1‐pHLIP@ [Cy5.5+BHQ3] NMs (peptide concentration of 0.5 mg mL^−1^) was incubated with MMP2 activating solution (2 µg mL^−1^) at a volume ratio of 1:1 for 0, 1, 2, 4, 8, or 12 h. A fluorescence spectrometer F4600 (excitation: 680 nm, emission: 710 nm) was used to measure Cy5.5 fluorescent intensity. The fluorescence quenching rate and recovery rate were calculated with the fluorescence value at 0 h as background.

To further assess the sensitivity of the fluorescence probe, different control probes with equal polypeptide concentration were prepared according to the same method above. Thus, the following experimental reagents and conditions were tested: PBS only (Control), preS1‐pHLIP@Cy5.5 NMs, preS1‐pHLIP@[Cy5.5+BHQ3] NMs, and preS1‐pHLIP@[Cy5.5+BHQ3] NMs + MMP2, which were incubated with MMP2‐activated liquid (2 µg mL^−1^) at a 1:1 volume ratio for 4 h. Cy5.5 fluorescence was read using a spectrometer. The fluorescence quenching rate and recovery rate were calculated using the control group as the background.

Evaluation of in vivo distribution and tumor targeting of preS1‐pHLIP NMs: SCC7 cells (2 × 10^6^ cells) were inoculated subcutaneously on the right dorsal side of 5‐week‐old BALB/C nude mice. When the tumors had grown to about 200 mm^3^, the mice were randomly divided into three groups. Before administration, preS1 peptide and PreS1‐pHLIP NMs were labeled with NHS‐Cy5.5. Then, SCC7 tumor bearing mice were injected with PBS and Cy5.5‐labeled preS1 and Cy5.5‐labeled preS1‐pHLIP NMs with the same amount of peptide (25 µM peptide per mouse) in the tail vein. After 6, 12, 24 and 36 h, the drug distribution in vivo was detected by IVIS Spectrum(PerkinElmer, USA). In order to test the sensitivity of preS1‐pHLIP in response to tumor MMP2 switching, 6‐week‐old C3H mice were subcutaneously inoculated with SCC7 cells (4 × 10^6^ cells) on the right dorsal side. When the tumor had grown to about 50 mm^3^, the mice were randomly divided into three groups. PreS1‐pHLIP@[Cy5.5+BHQ3] NMs, a fluorescent probe prepared according to the above method was used to monitor the release of preS1‐pHLIP NMs in vivo. PBS, free Cy5.5 and preS1‐pHLIP@ [Cy5.5+BHQ3] NMs were injected intravenously into tumor‐bearing mice, and the fluorescence distribution was detected by in vivo imaging after 6, 12, 24 and 36. In addition, in vitro imaging analysis was performed on heart, liver, spleen, lung, kidney and tumor from mice euthanized 12 h after injection. Fluorescence intensity was analyzed by Living Image system.

### Immunofluorescence Staining

Immunofluorescence of cells: SCC7 cells were seeded on poly‐L‐lysine‐pretreated coverslips (Cellvis, USA, Cat: 05 1410). When cells reached ≈70% confluence, 20 µM preS1‐pHLIP was added an incubated for 1, 2, 4, 8, 12 and 24 h at 37 °C, pH 6.8. The cells were then fixed with cold methanol for 10 min and washed twice. The coverslips were incubated with 50 µg mL^−1^ anti‐PreS1 (1:1000) in PBS for 1 h at 37 °C. After three washes with PBS, Alexa Fluor 594‐conjugated anti‐mouse IgG was incubated at 37 °C for 2 h, and the nuclei were stained with Hoechst 33 342 (Invitrogen, USA, Cat: H3570). The coverslips were mounted on slides with Fluoroshield (Sigma, Cat: F4680) and imaged with a Laser Scanning Confocal Microscopy (LSCM, Nikon Eclipse Ti2).

Immunofluorescence staining of tumor tissue: after the mouse tumor was collected, tumor tissues of 1.0 cm × 1.0 cm × 0.5 cm were placed into an embedded box equipped with optimal cutting temperature compound (OCT), infiltrated and frozen. The tissues were then cut into 8‐µm thick slices in a cryomicrotome, harvested on pre‐treated slides, and stored at −20 °C until use. Before immunofluorescence staining, the sections were thawed and fixed with 4% paraformaldehyde and soaked in PBS to remove OCT. The sections were blocked with 10% goat serum at room temperature for 1 h. Next, the sections were incubated with the anti‐preS1 antibody at 4 °C overnight. The next day, the slides were washed with PBS, and incubated with the Alexa Fluor594‐conjugated anti‐mouse IgG at 37 °C for 2 h. After washing in PBS, the nuclei were stained with Hoechst 33 342 (Invitrogen, Cat: H3570). Finally, the coverslips were mounted with Fluoroshield, and fluorescent images were captured using a LSCM as above.

### Antibody Titration by ELISA

To detect PreS1‐specific antibody titers, 5 µg mL^−1^ preS1_(38‐47)_ peptide were coated onto 96‐well high‐binding assay plates in ELISA coating buffer (Solarbio, Cat: C1055) at 4 °C overnight. After blocking with 5% skim milk for 5 h, serial‐dilutions of the serum samples were added to the plate and incubated at 4 °C overnight after milk was removed. After washing five times with PBST (PBS + 0.05% Tween 20), Horse Radish Peroxidase (HRP)‐conjugated goat anti‐mouse IgG (H+L) (1:5000, Bio‐Rad, USA, Cat: 1 706 516) was added and incubated at 37 °C for 1 h. HRP was detected using the chromogenic substrate 3,3′,5,5′‐Tetramethylbenzidine (TMB; Solarbio, Cat: PR1200), and the absorbance at 450 nm was measured using an enzyme labeler (TECAN Spark) after the reaction was terminated by Stop Solution (Solarbio, Cat: C1058). The absolute concentration of anti‐preS1 IgG in serum was obtained using a mouse anti‐preS1 of known concentration as a standard. The quantity of preS1 antibody per sample was calculated by comparing the standard curve to serum IgG concentration.

### Evaluation of the Antitumor Effect

Anti‐HNSCC tumor evaluation: 6‐week‐old C3H mice were randomly divided into 7 groups as follows: no pre‐immunity + PBS, no pre‐immunity + preS1, no pre‐immunity + NMs, pre‐immunity + PBS, pre‐immunity + preS1, pre‐immunity +Random NMs, pre‐immunity + preS1‐pHLIP NMs. Mice were pre‐immunized or not by intramuscular injection of preS1(38‐47) peptide three times at 2‐week intervals. 5 days after the last immunization, SCC7 cells (4 × 10^6^ cells each) were inoculated subcutaneously, and treatment was initiated when the tumor had grown to 50 mm^3^ (about 5 days after inoculation). Treatment consisted of four intravenous administrations at 4‐day intervals, at a dose of 5 µM peptide/per mouse (50 µM NMs, 100 µL per mouse). Tumor growth was monitored from the first dose (measured every other day). At day 20 after beginning of treatment, some mice were sacrificed, and the tumors were analyzed for local immune responses, while the remaining mice were monitored for survival. For survival, the mice were sacrificed when the tumor reached 1000 mm^3^.

Anti‐ATC tumor evaluation: 6‐week‐old C57 mice were pre‐immunized by intramuscular injection of preS1 peptide three times at 2‐week intervals, and then were randomly divided into 4 groups as follows: pre‐immunity + PBS, pre‐immunity + Random NMs, pre‐immunity + preS1, pre‐immunity + preS1‐pHLIP NMs. 5 days after the last immunization, mATCs cells (2 × 10^6^ cells each) were inoculated subcutaneously, and treatment was initiated when the tumor had grown to 50 mm^3^. Treatment consisted of four intravenous administrations at 4‐day intervals, at a dose of 5 µM peptide/per mouse (50 µM NMs, 100 µL per mouse). The first injection was recorded as day 0, and treatments were performed every 4 days. Tumor volumes were measured every other day until day 14. On day 16, the mice were euthanized for tumor photography, weighing, and immune analysis.

Anti‐recurrent HNSCC tumor evaluation: 6‐week‐old C3H mice were pre‐immunized three times as above, and SCC7 cells (4 × 10^6^ cells each) were inoculated subcutaneously 5 days after the last injection. When the tumor had grown to about 200 mm^3^ (about 9 days after inoculation), it was removed and sutured, and then the mice were divided into the following 4 groups: pre‐immunity + PBS, pre‐immunity + preS1, pre‐immunity + Random NMs, pre‐immunity + preS1‐pHLIP NMs. Then, mice were treated intravenously at days 4, 8, 12 and 16 post surgery. At day 20 after beginning of treatment, the mice were sacrificed, and the tumor was photographed.

Anti‐HNSCC lung metastasis evaluation: 6‐week‐old C3H mice were pre‐immunized three times as above. 5 days after the last injection, SCC7 cells (2 × 10^6^ cells each) were injected into the tail vein. The mice were then divided into the following four groups: pre‐immunity + PBS, pre‐immunity + preS1, pre‐immunity + Random NMs, pre‐immunity + preS1‐pHLIP NMs. Then, mice received the different treatments intravenously at days 4, 8, 12 and 16 after tumor injection. At day 20 after beginning of treatment, the mice were sacrificed, the lung tissues were taken, and the number of lung metastases was counted.

### Immune Response Analysis

For the analysis of the immune cell populations in the tumor, the tumor tissue was harvested, cut into pieces in digesting buffer (containing 0.05% pancreatic enzyme, 1 mg mL^−1^ collagenase D, 20 µg mL^−1^ DNase I), and incubated in a shaker at 100 rpm min^−1^ and 37 °C for 30 min. The digestion was terminated by addition of 1640 RPIM medium containing 10% FBS, and the suspensions was filtered through a 70‐µm cell strainer. The cells were washed twice in 1640 RPIM containing 2% FBS to obtain a single‐cell suspension, which was split to perform various assays to quantify B cells, antigen‐specific B cells (CD19^+^ cells), T cells (CD3^+^ cells), CD8^+^ T cells (CD3^+^CD8^+^ cells), macrophages (F4/80^+^ cells), DC cells (CD11C^+^ cells), and neutrophils (CD11B^+^Ly6G^+^) using flow cytometry. For antigen‐specific B cells, preS1 peptide was pre‐labeled with NHS‐Cy5.5 (APExBIO, USA, Cat: #A8103).

Tumor antigen‐specific T cells immunoassay: single cell suspension was obtained from mouse splenocytes after grinding through a 70‐µm cell strainer and treatment with red cell lysing buffer for 3 min. after centrifugation the splenocytes were washed with PBS, and then cultured with DMEM containing 10% FBS. SCC7 cell lysate (10 µg mL^−1^) was added to the splenocytes for overnight stimulation, and then IFN‐γ^+^ cells among the CD3^+^CD8^+^ T cells and the CD3^+^CD4^+^ T cells were quantified by flow cytometry.

### ADCC, ADCP and CDC Evaluation

The ADCC experiment was performed as previously described.^[^ ^11^ ^]^ Briefly, primary NK cells were labeled with CD49B and sorted from mouse spleens by flow cytometry. Purified NK cells were cultured with IMDM medium containing 500 U mL−1 recombinant mouse IL‐2 for 8 days to generate lymphokine‐activated killer (LAK) cells. SCC7 cells (8000 cells per well) were incubated with PBS, 20 µM free preS1 peptide, preS1‐pHLIP NMs or preS1‐pHLIP NMs + MMP2 in 96‐well plates for 2 h (pH = 6.8). Next, the pre‐treated SCC7 cells were incubated with immunized mouse serum (dilution: 1:100) at 37 °C for 2 h. The cells were then washed, seeded in 96‐well plates (2000 cells per well), and incubated with LAK cells (2000 cells per well). After 12 h, the culture supernatant was collected and LDH release was measured (Beyotime Biotechnology, China, Cat: C0016).

For ADCP evaluation, BMDMs were prepared and cultured as previously described.^[^ ^12^ ^]^ SCC7 cells (8000 cells per well) were incubated with 20 µM free preS1 peptide, preS1‐pHLIP NP or preS1‐pHLIP NMs + MMP2 in 96‐well plates for 2 h (pH = 6.8). The pre‐treated SCC7 cells were labeled with 3,3′‐Dioctadecyloxacarbocyanine perchlorates (DIO; MedChemExpress, Cat: HY‐D0969), and then mouse serum diluted in 50 µL medium (1:50) was added and incubated at 37 °C for 30 min. The treated SCC7 cells were then incubated with BMDMs for 6 h, and adherent cells were collected. The proportion of DIO^+^ cells among the F4/80+ cells represented a readout of the percentage of tumor cells engulfed by the BMDMs.

CDC evaluation: SCC7 cells (8000 cells per well) were incubated with 20 µM free preS1 peptide, preS1‐pHLIP NMs, or preS1‐pHLIP NMs + MMP2 in 96‐well plates for 2 h (pH = 6.8). Next, mouse serum diluted in 50 µL RPMI culture medium (1:40) was added and incubated at 37 °C for 30 min. Cavy sera complement diluted with 50 µL RPMI culture medium (1:5 dilution, Yuanye Bio‐Technology, Cat: S27068, China) was then added and incubated at 37 °C for 4 h. Cell viability was measured by colorimetric method, using 3‐(4,5‐dimethylthiazol‐2‐yl)‐5‐(3‐carboxymethoxyphenyl)‐2‐(4‐sulfophenyl)‐2H‐tetrazolium (MTS; Promega, USA, Cat: G3582).

### Statistical Analysis

Statistical analysis was performed using GraphPad Prism 8, and data are presented as mean ± standard deviation (SD). At least three independent experiments were performed for statistical analysis, the number of samples in different experiments are shown in the figure legends. P values for two‐group comparisons were determined using Student's t‐tests. P values for multiple‐group comparisons, one‐way ANOVA followed by Tukey's test was applied. *P < 0.05, **P < 0.01 and ***P < 0.001 were considered statistically significant. Kaplan‐Meier survival analysis was evaluated using Log‐rank test.

## Conflict of Interest

The authors declare no conflict of interest.

## Author contributions

L.Z., Q.F., C.Z., and Y.L. contributed equally to this work. M.G., Q.L., and K.C. designed the research. L.Z., Q.F., X.G., Y.L., Z.Y., Z.T., T.J., J.W., C.Z., J.X., H.G., T.J., W.D., X.Z., and K.C. performed the research. All authors analyzed and interpreted the data. L.Z., Q.F., X.G., and K.C. wrote the paper.

## Data availability

The main data supporting the results in this study are available within the paper and its Supplementary Information. There is no data from third‐party or publicly available datasets.

## Supporting information



Supporting Information
