# Supplementary material for: Antigen‐Targeting Inserted Nanomicelles Guide Pre‐Existing Immunity to Kill Head and Neck Cancer
Source: Adv Sci (Weinh). 2025 Mar 17;12(18):2410629. doi: 10.1002/advs.202410629 (PMC12079393; doi:10.1002/advs.202410629)
Supplement: Supplementary file 1 — Supporting Information [file ADVS-12-2410629-s001.docx]

**Supplementary Information**

**Antigen-Targeting Inserted Nanomicelles Guide Pre-Existing Immunity to Kill Head and Neck cancer**


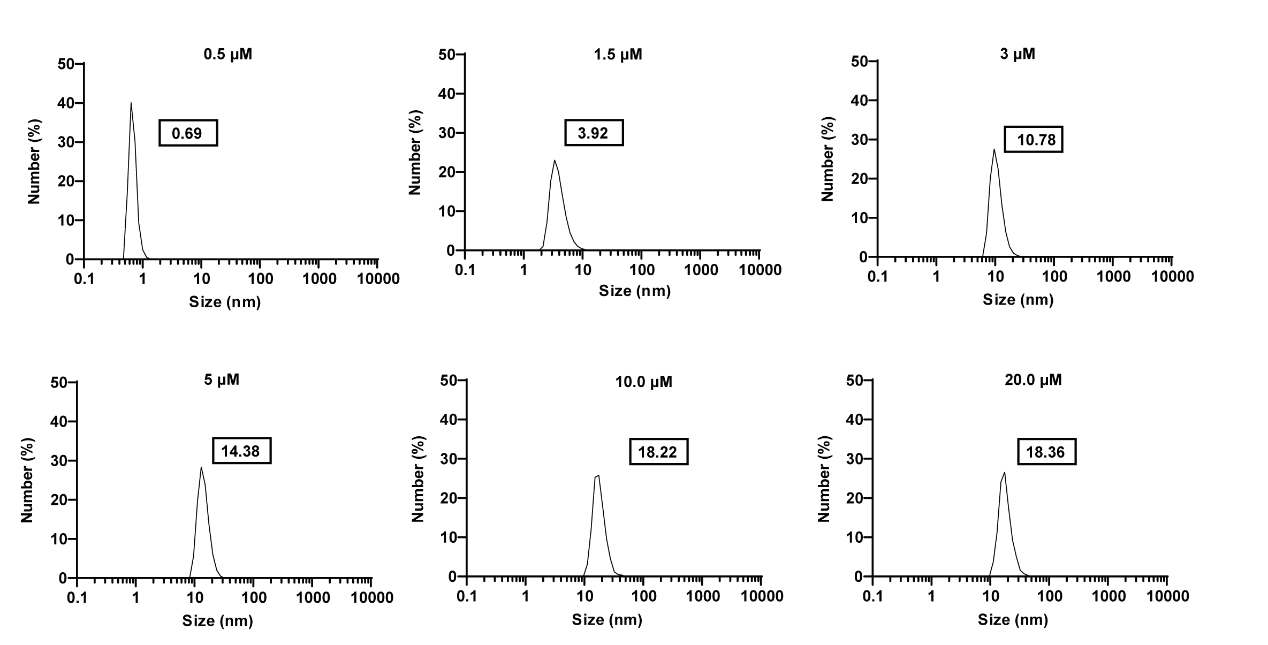
**Supplementary Figure 1.** DLS analysis of PEG-PLG-PreS1-pHLIP with different concentrations after resting on PBS (PH=6.8) for 30 minutes. PEG-PLG-PreS1-pHLIP was prepared with PBS (PH=6.8) for 0.5 μM, 1.5 μM, 3 μM, 5 μM, 10 μM and 20 μM. The particle size of each group was analyzed after standing at room temperature for 30 min (n=3).


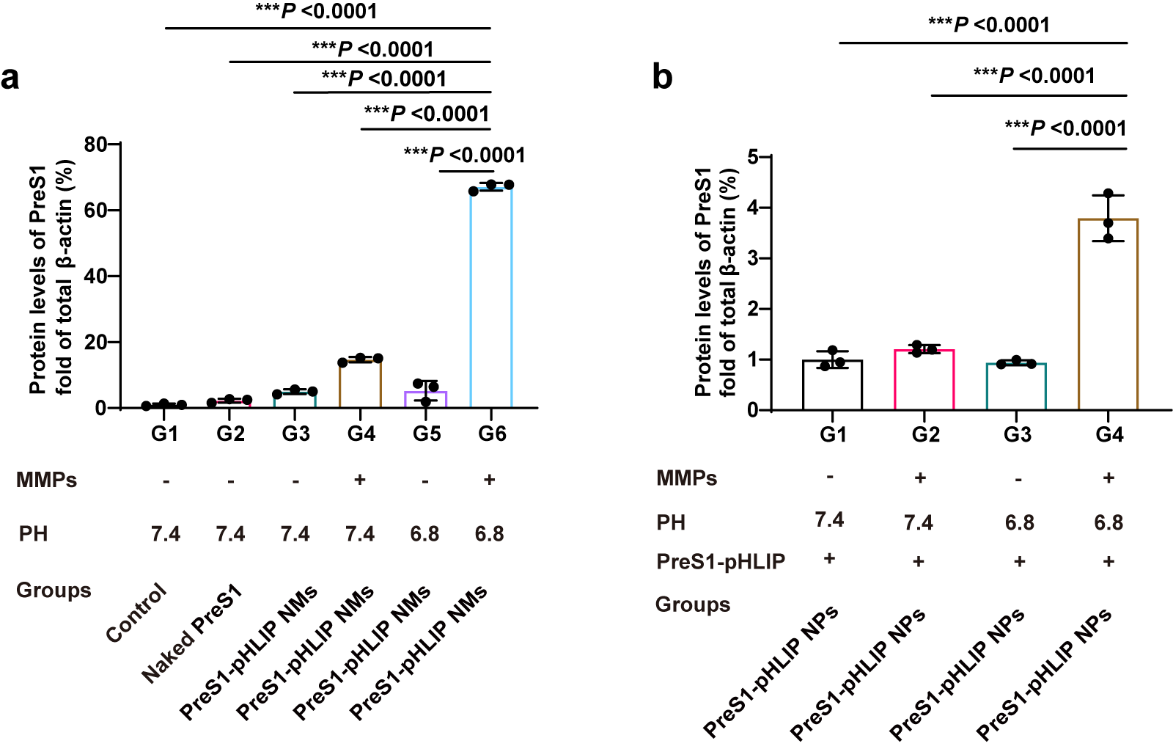
**Supplementary Figure 2.** Analysis of gray of western blotting showed that PreS1 content on the cell membrane of SCC7 at PH=7.4 or PH=6.8 with or without MMP2 treatment. (a) Analysis of gray of figure 2g (n=3). (b) Analysis of gray of figure 2b (n=3). Statistical significance (P value) was calculated were determined using one-way ANOVA with a Tukey post-hoc test. ***, *P* < 0.001.


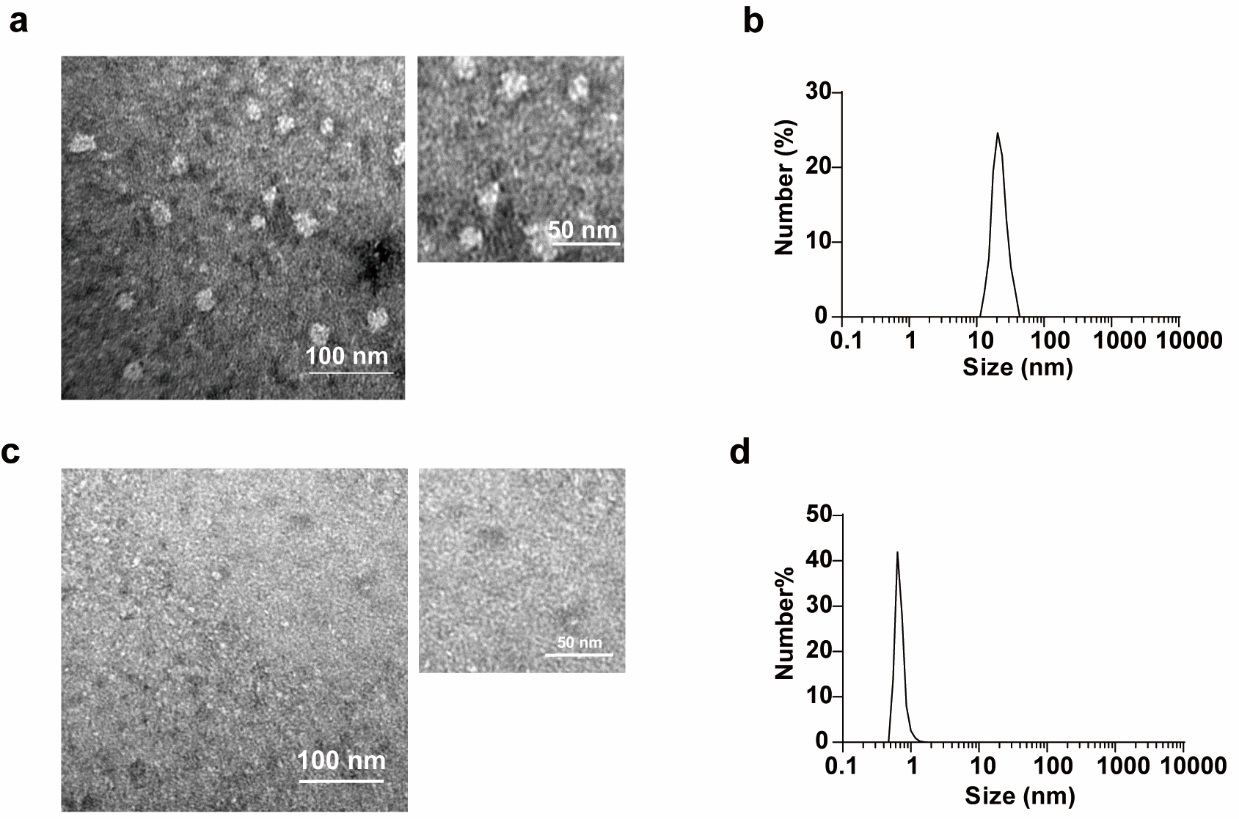


**Supplementary Figure 3.** Characterization of self-assembled peptide nanomicelles labeled with Cy5.5. (a) Particle size and (b) TEM images of Cy5.5-labeled NMs. (c) Particle size and (d) TEM images of Cy5.5-labeled NMs treated with MMP2.


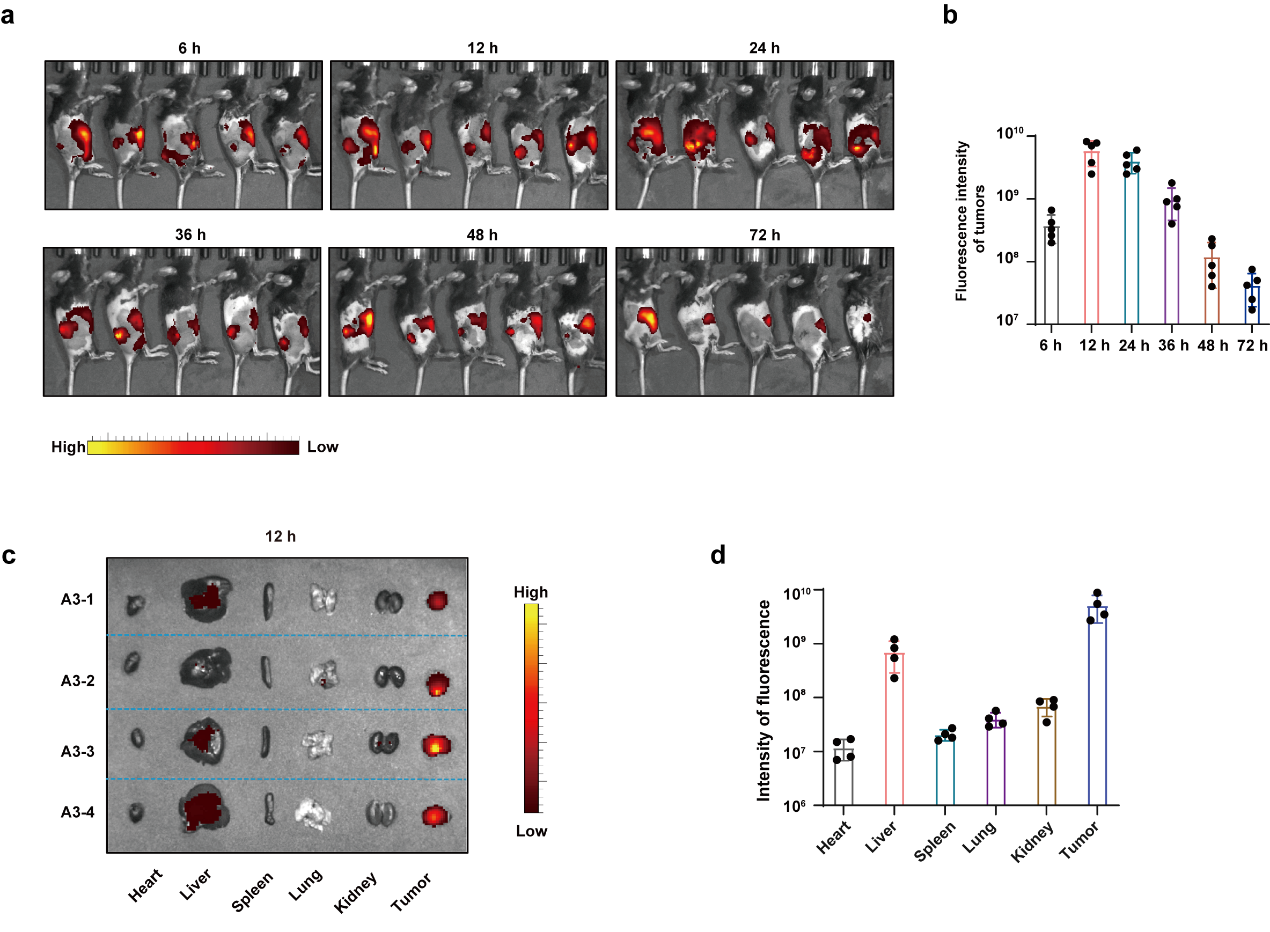


**Supplementary Figure 4.** Distribution of preS1-pHLIP NMs in the mouse. (a) Mouse images and (b) statistical summary of the fluorescence kinetics in subcutaneous SCC7 tumor-bearing mice injected intravenously with preS1-pHLIP@[Cy5.5+BHQ3] NMs (group 3, A3), and measured at 6, 12, 24, 36 h, 48 h and 72 h post-injection (n = 5 mice/group). (c) Main tissues and tumor images, and (g) statistical summary of the fluorescence kinetics in subcutaneous SCC7 tumor-bearing mice injected intravenously with preS1-pHLIP@[Cy5.5+BHQ3] NMs (group 3, A3), and measured at 12 h post-injection (n = 4 mice/group).


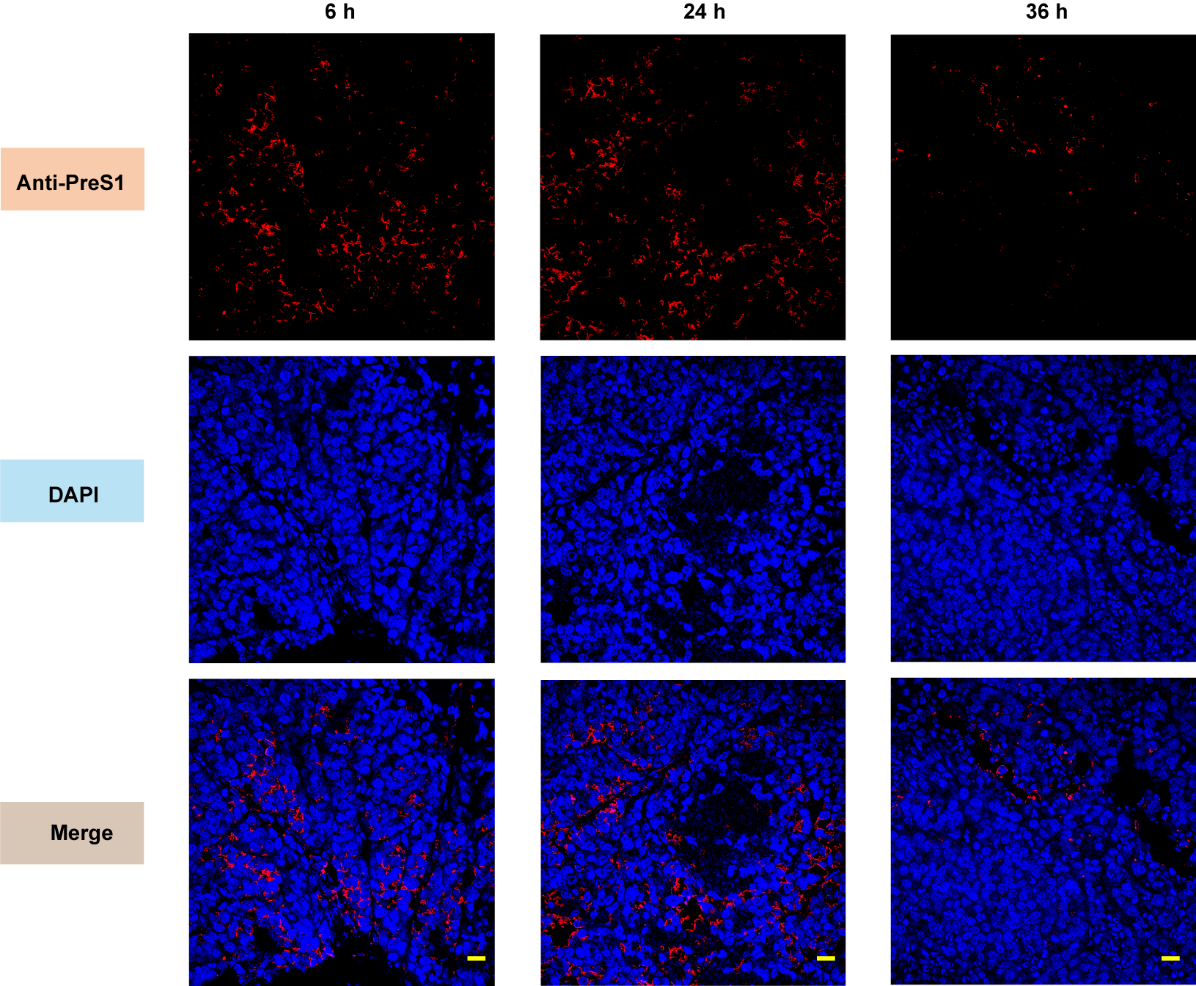
**Supplementary Figure 5.** Representative immunofluorescence images of PreS1^+^ cells in tumor tissues after preS1-pHLIP NMs injection for 6 h, 24 h and36 h. Scale bar, 20 μm.


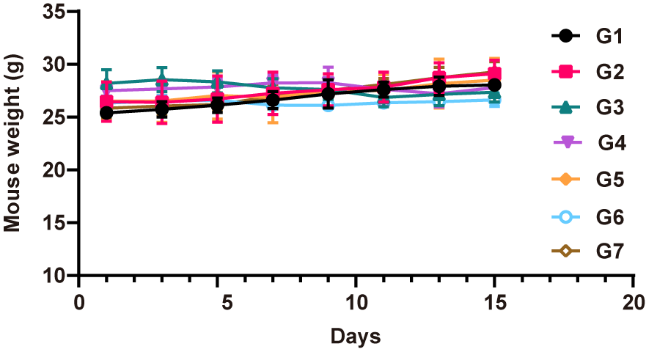


**Supplementary Figure 6.** Body weight statistics of mice during the experimental period of antitumor therapy. The experimental grouping information is shown in Figure 4f.


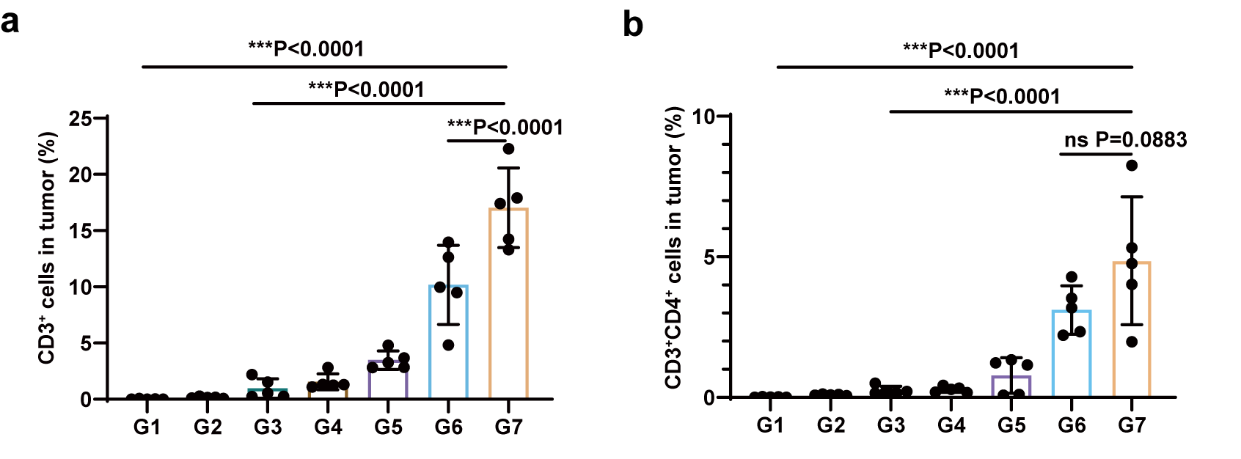


**Supplementary Figure 7.** Proportions of CD3^+^ (a) and CD3^+^CD4^+^ (b) T cells in tumor tissues (n = 5). The experimental schedule and grouping information are shown in Figure 4f. ***, *P* < 0.001; ns, no significant difference.


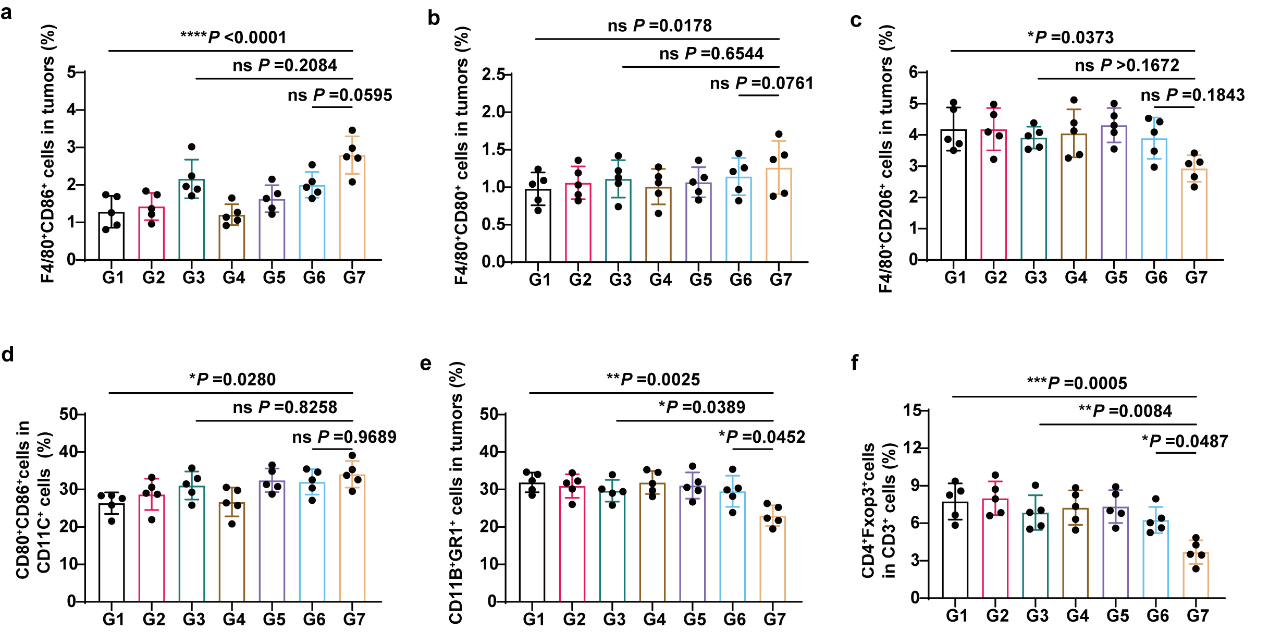


Supplementary Figure 8. Immune cell infiltrates induced by PreS1-pHLIP NMs within the mice tumors were analyzed by flow cytometry on day 20 after the beginning of treatment. (cf. Figure 5). Proportions of (a) F4/80+CD86+ M1-polarized macrophages, (b) F4/80+CD80+ M1-polarized macrophages, (c) F4/80+CD206+ M2-polarized macrophages, (d) CD80+CD86+ cells in CD11C+ cells, (e) CD11B+GR1+ MDSCs, and (f) CD4+Foxp3+ Treg cells in CD3+ T cells in tumor tissues (n = 5). Statistical significance (P value) was calculated using one-way ANOVA with a Tukey post-hoc test. *, *P* < 0.05; ****, *P* < 0.01; ***, P < 0.001. ns, P > 0.05, no significant difference.


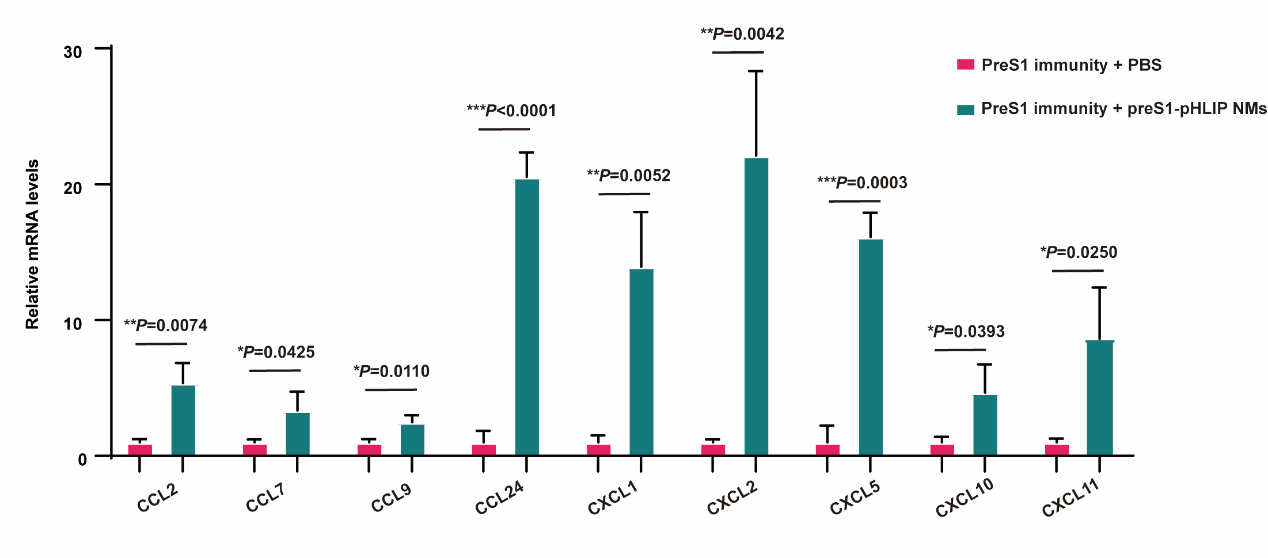


**Supplementary Figure 9.** Relative mRNA levels of chemokines CCL2, CCL7, CCL9, CCL24, CXCL1, CXCL2, CXCL5, CXCL10 and CXCL11in tumor tissues (n = 3). Statistical significance (P value) was calculated were determined using unpaired t-test. ***, *P* < 0.05; **, *P* < 0.01; ***, *P* < 0.001.


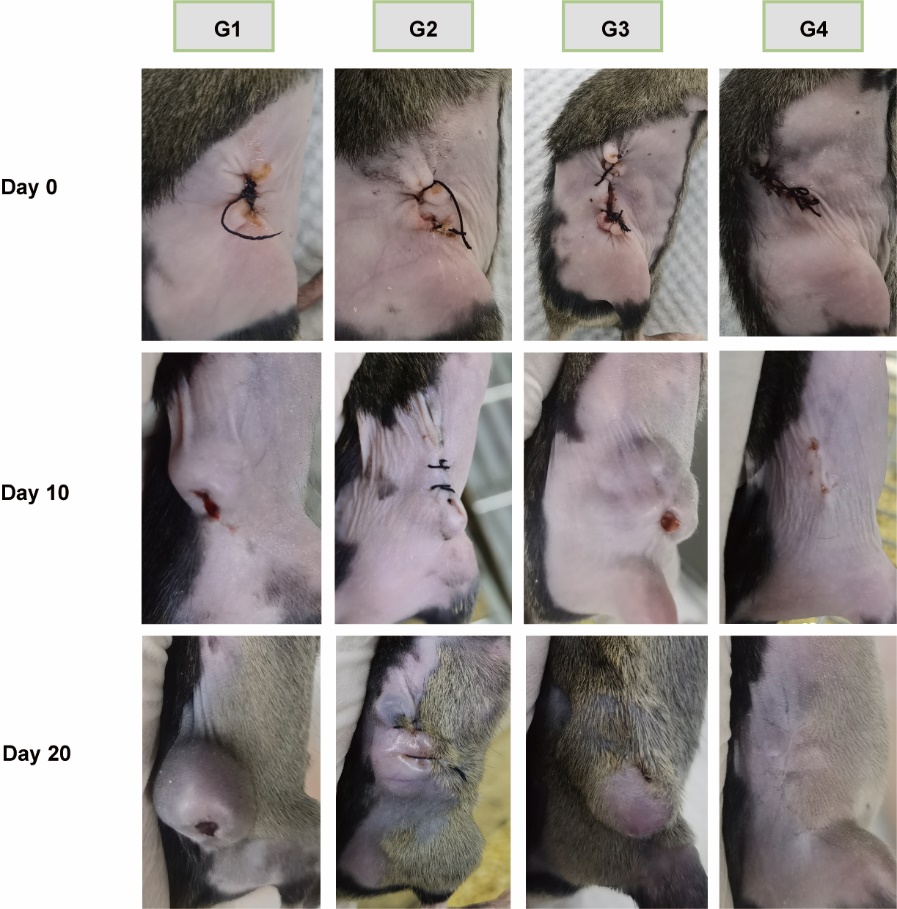


**Supplementary Figure 10.** Representative image of tumor-bearing mice in postoperative recurrent HNSCC animal model. The experimental schedule and grouping information are shown in Figure 6b.


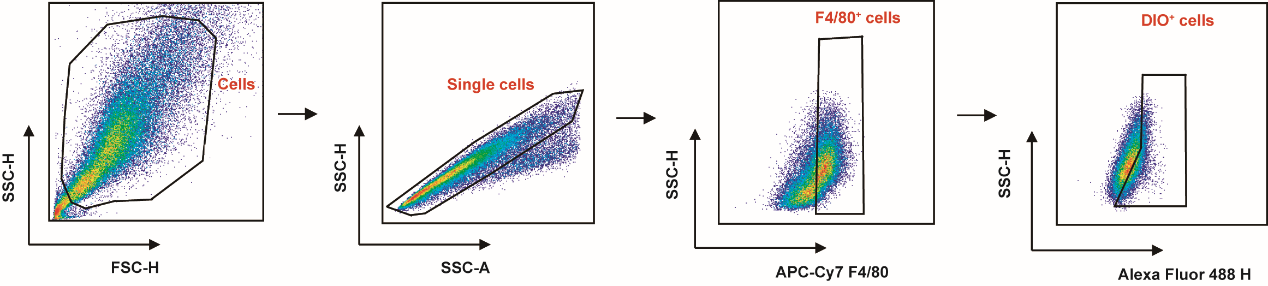


**Supplementary Figure 11.** Gating strategy for Figure 4d.


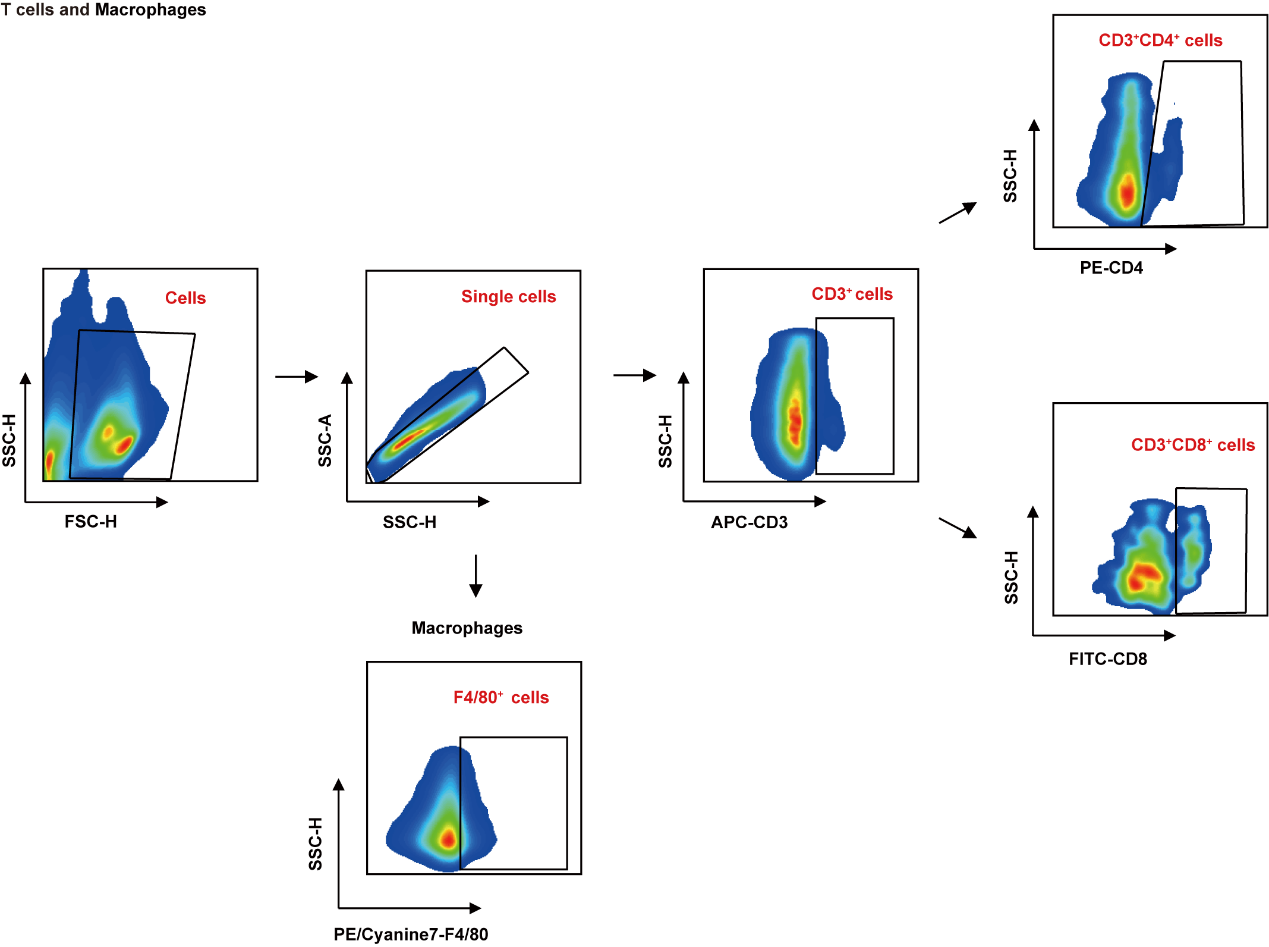


**Supplementary Figure 12.** Gating strategy for Figure 5c, e and Supplementary Figure 2.


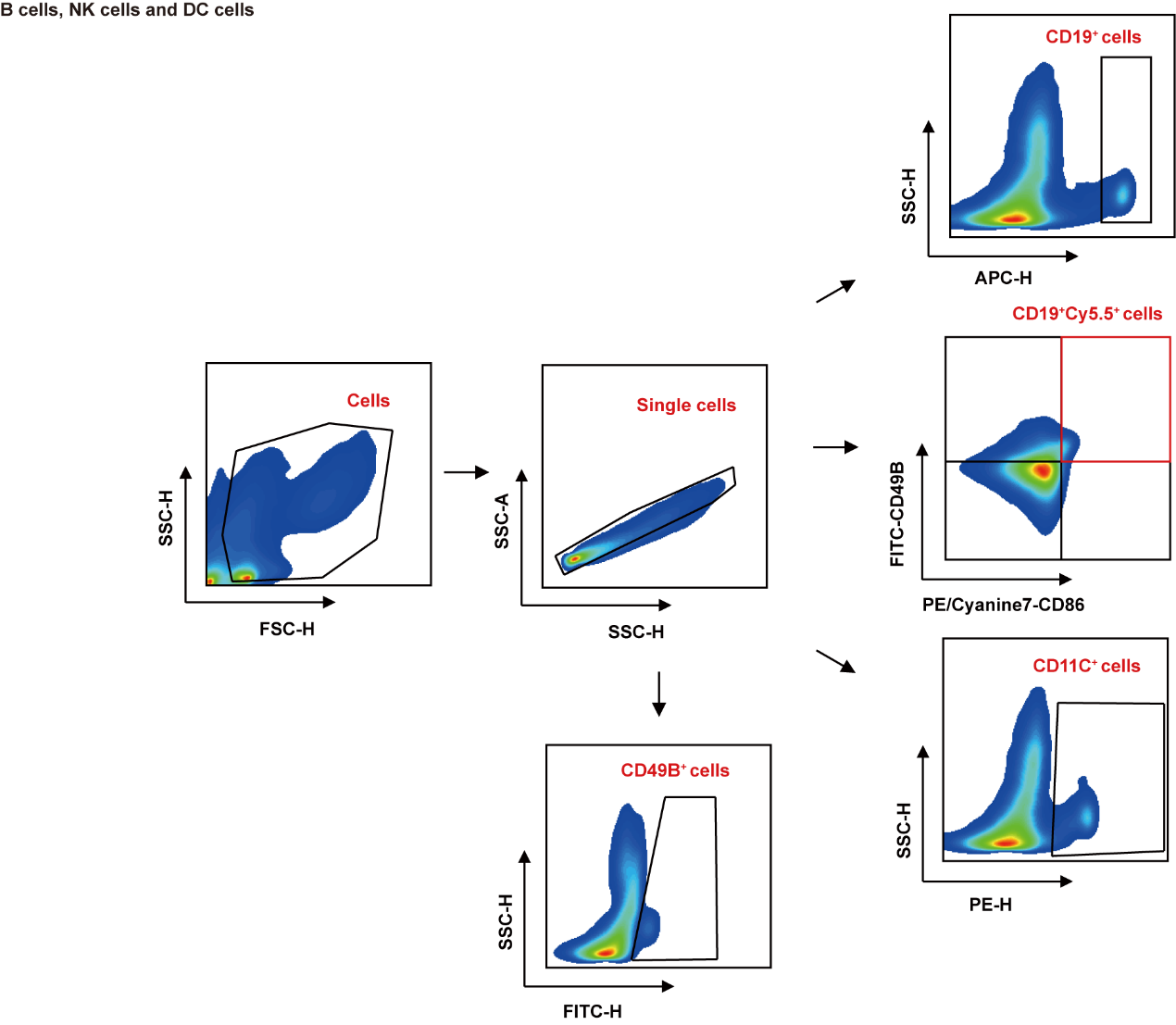


**Supplementary Figure 13.** Gating strategy for Figure 5a, b, d, f.


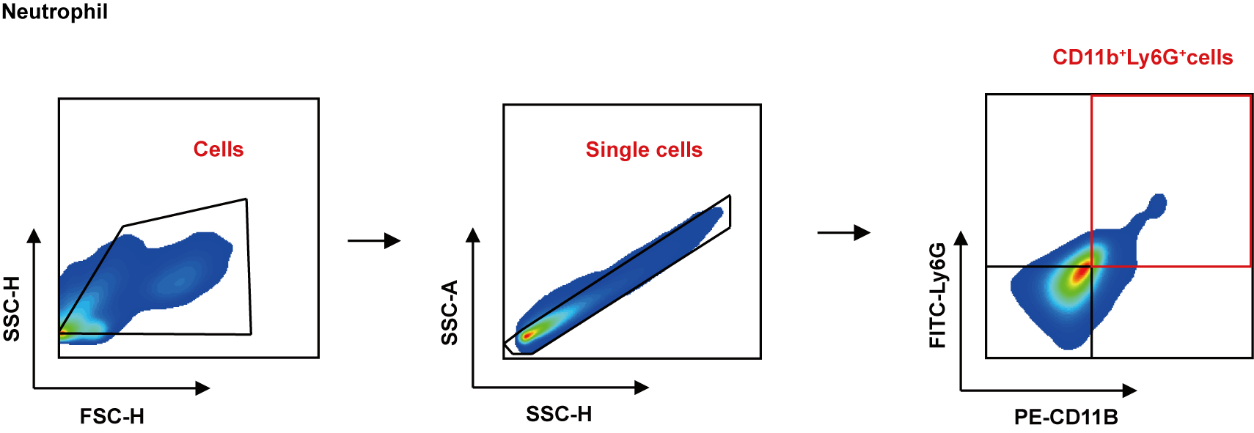


**Supplementary Figure 14.** Gating strategy for Figure 5g.


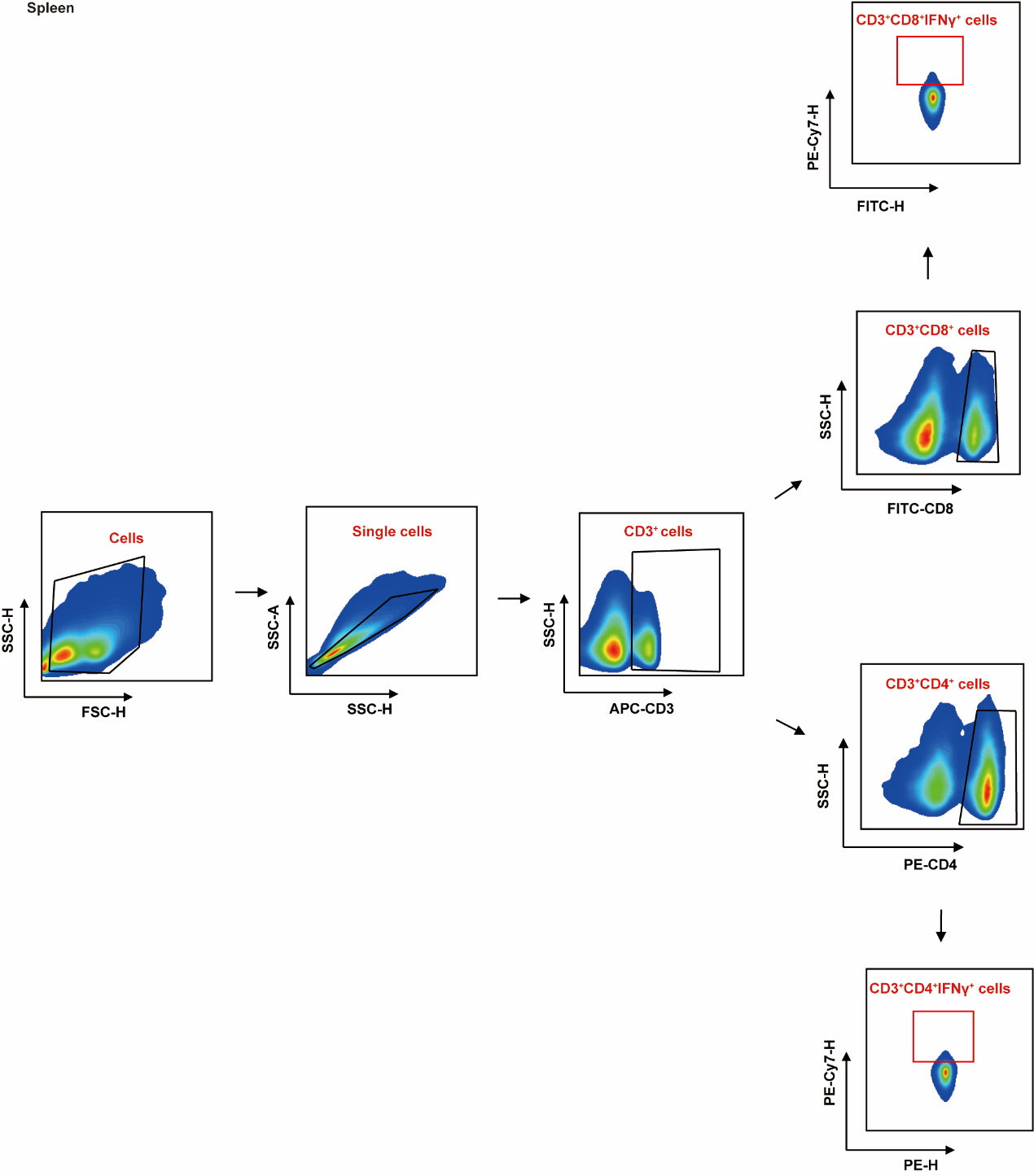


**Supplementary Figure 15.** Gating strategy for Figure 5h, i.


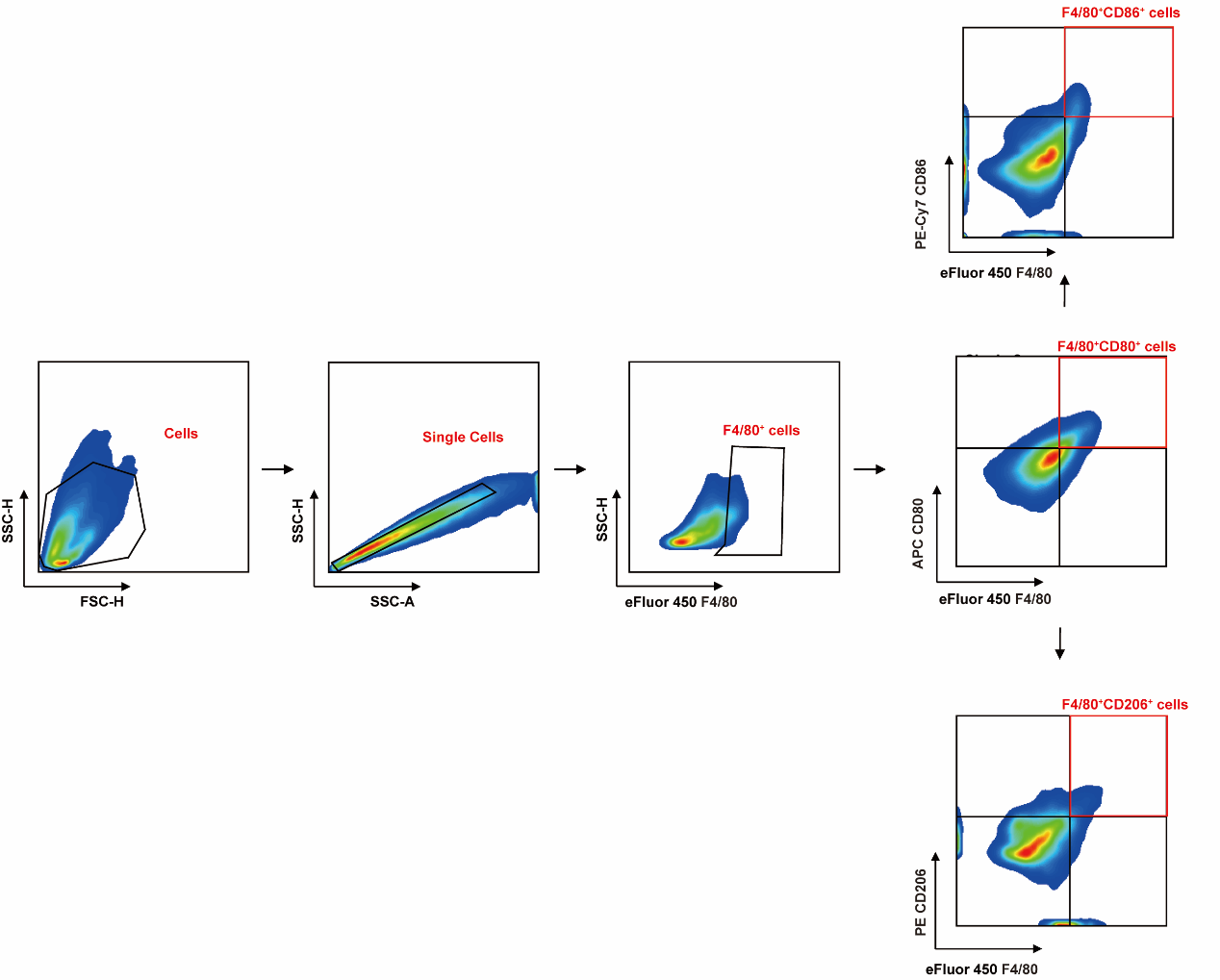


**Supplementary Figure 16.** Gating strategy for Figure 8a, b, c.


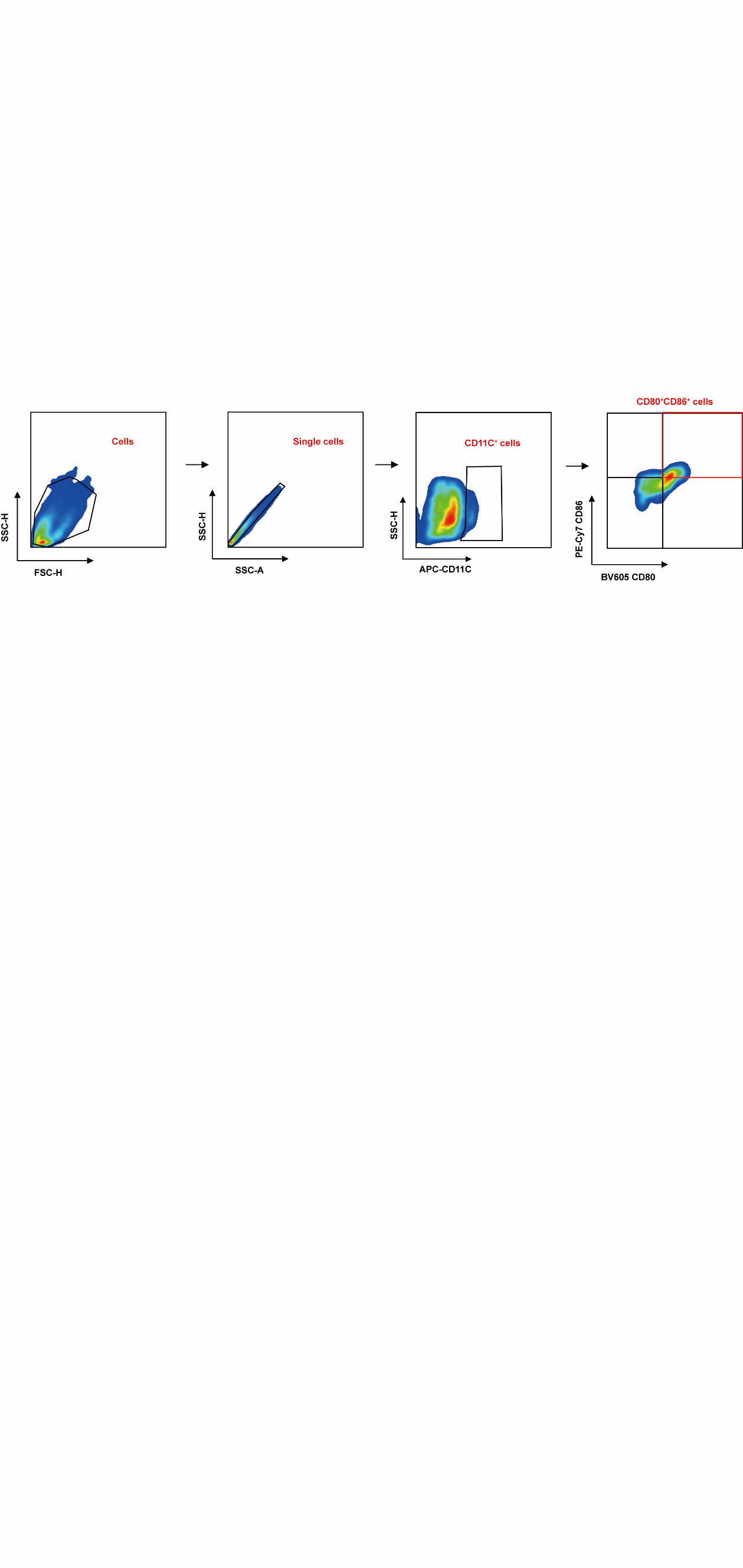


**Supplementary Figure 17.** Gating strategy for Figure 8d.


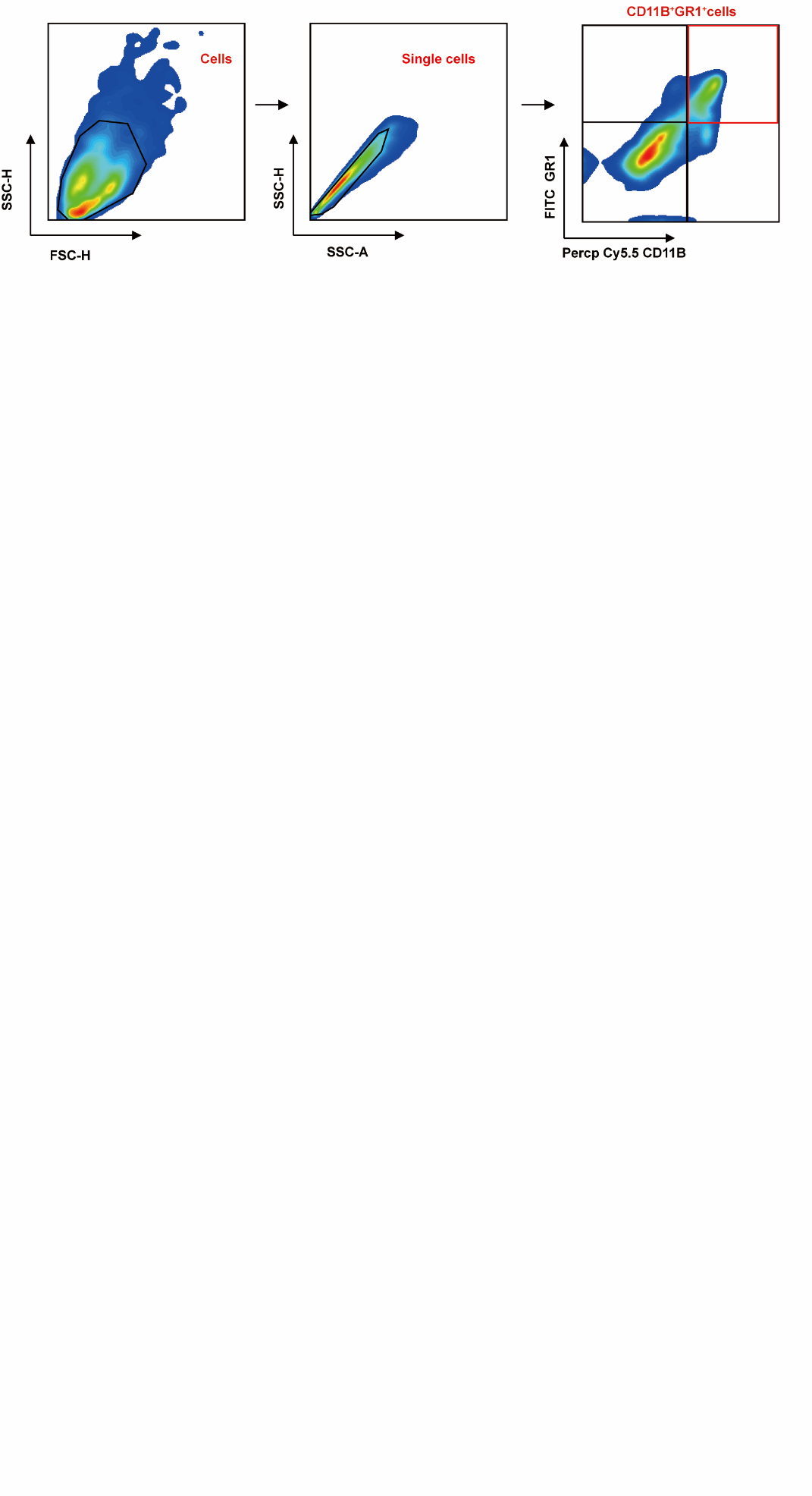


**Supplementary Figure 18.** Gating strategy for Figure 8e.


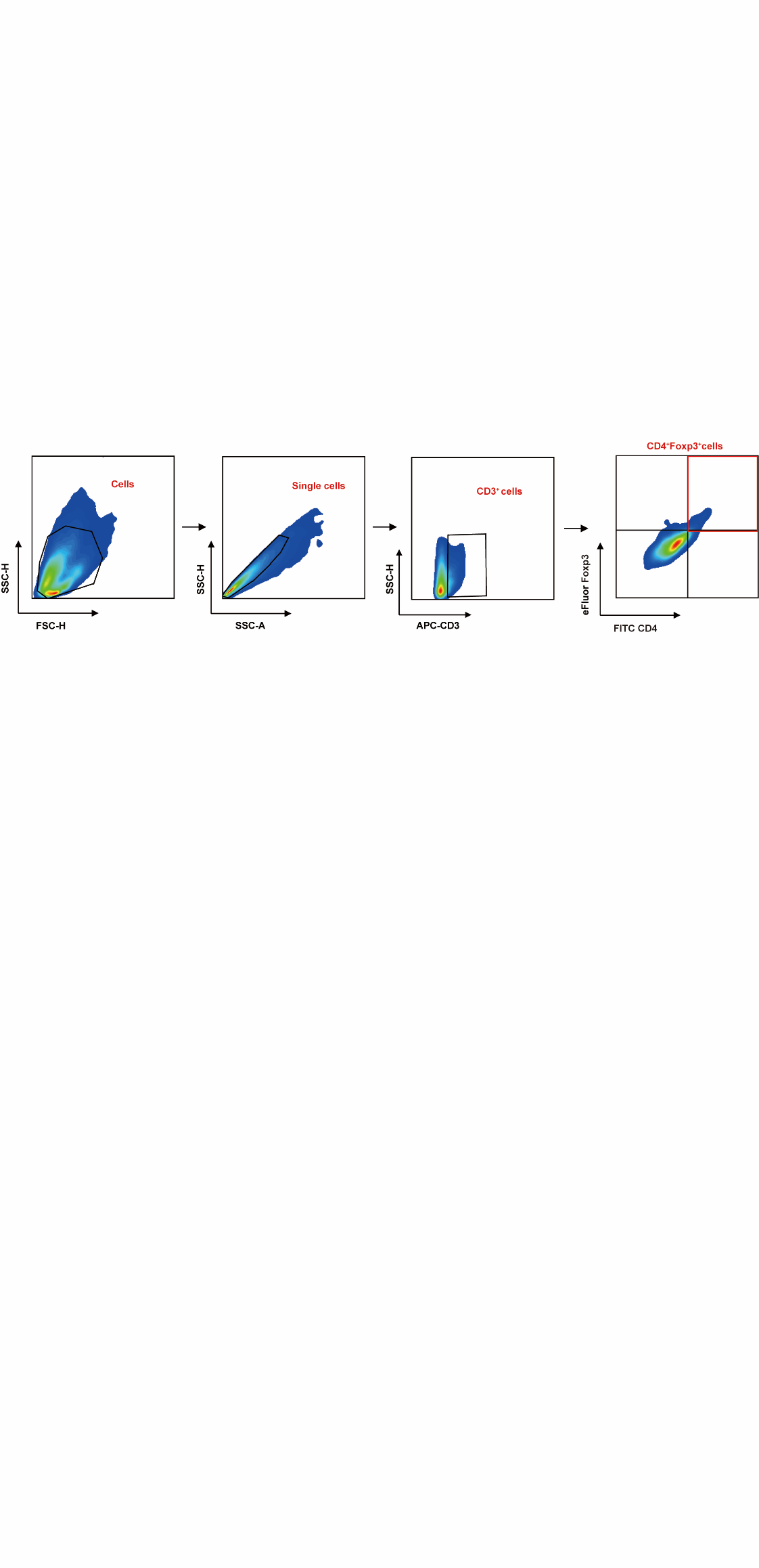


**Supplementary Figure 19.** Gating strategy for Figure 8f.


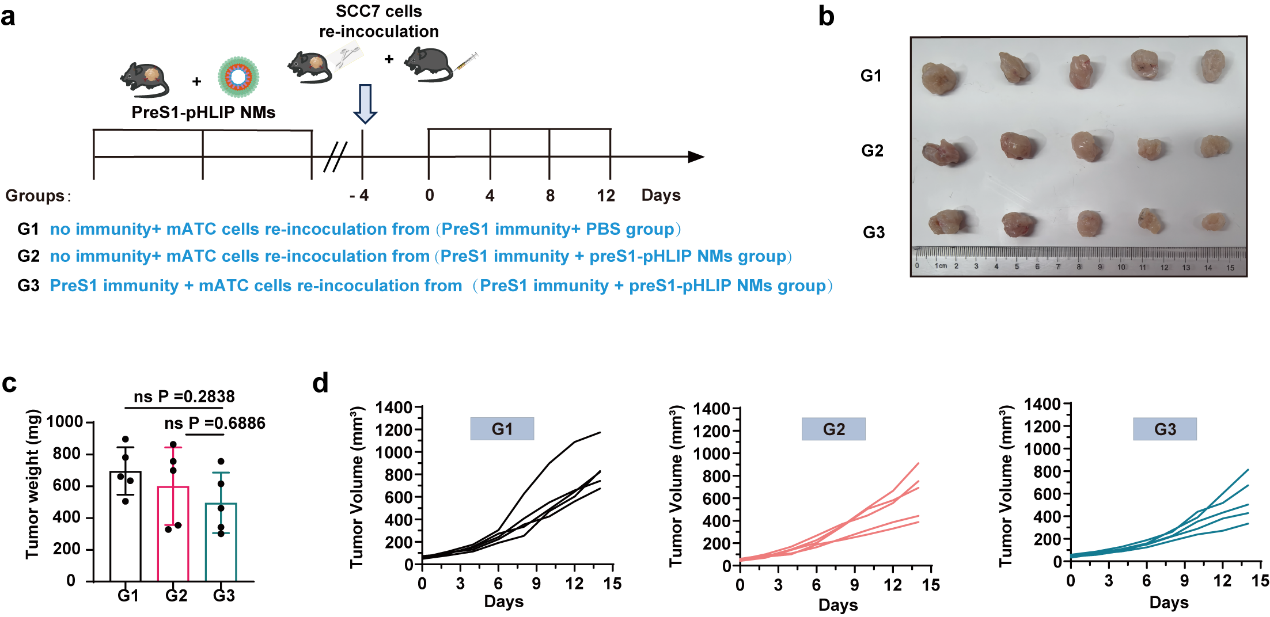


**Supplementary Figure 20.** Anti-tumor evaluation against cancer-initiating cells. Tumor tissues from groups G1 and G7, treated as described in Figure 4, were harvested, minced, and digested, then transplanted into naive (non-preimmunized) and preimmunized C3H mice. Subsequent tumor growth was observed and recorded. (a) Schematic illustration of the experiment schedule and group identification/key. (b) Tumor image, (c) tumor weight, and (d) growth curves of subcutaneous tumors in each mouse. (n = 5). Statistical significance (P value) was calculated using one-way ANOVA with a Tukey post-hoc test. ns, no significance.
